# Sequence Control of the Self-Assembly of Elastin-Like Polypeptides into Hydrogels with Bespoke Viscoelastic and Structural Properties

**DOI:** 10.1021/acs.biomac.2c01405

**Published:** 2022-12-14

**Authors:** Diego López Barreiro, Abel Folch-Fortuny, Iain Muntz, Jens C. Thies, Cees M.J. Sagt, Gijsje H. Koenderink

**Affiliations:** † DSM Biosciences and Process Innovation, DSM, Alexander Fleminglaan 1, 2613 AXDelft, The Netherlands; ‡ DSM Biodata and Translation, DSM, Alexander Fleminglaan 1, 2613 AXDelft, The Netherlands; § Department of Bionanoscience, Kavli Institute of Nanoscience Delft, Delft University of Technology, Van der Maasweg 9, 2629 HZDelft, The Netherlands; ∥ DSM Biomedical, DSM, Urmonderbaan 22, 6160 BB, Geleen, The Netherlands

## Abstract

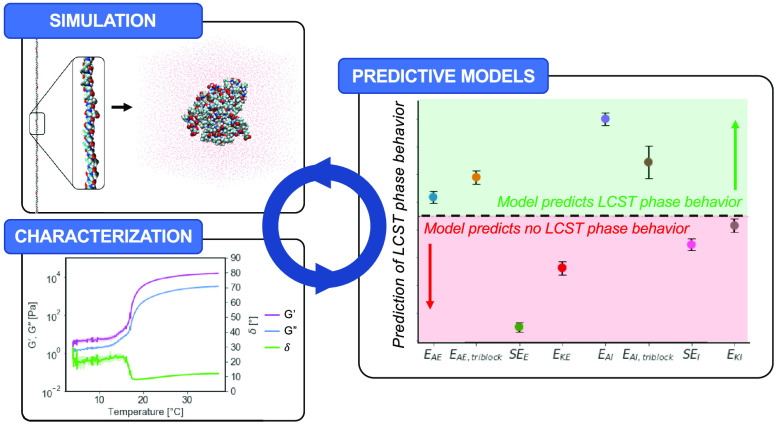

The biofabrication of structural proteins with controllable properties via amino acid sequence design is interesting for biomedicine and biotechnology, yet a complete framework that connects amino acid sequence to material properties is unavailable, despite great progress to establish design rules for synthesizing peptides and proteins with specific conformations (e.g., unfolded, helical, β-sheets, or β-turns) and intermolecular interactions (e.g., amphipathic peptides or hydrophobic domains). Molecular dynamics (MD) simulations can help in developing such a framework, but the lack of a standardized way of interpreting the outcome of these simulations hinders their predictive value for the design of *de novo* structural proteins. To address this, we developed a model that unambiguously classifies a library of *de novo* elastin-like polypeptides (ELPs) with varying numbers and locations of hydrophobic/hydrophilic and physical/chemical-cross-linking blocks according to their thermoresponsiveness at physiological temperature. Our approach does not require long simulation times or advanced sampling methods. Instead, we apply (un)supervised data analysis methods to a data set of molecular properties from relatively short MD simulations (150 ns). We also experimentally investigate hydrogels of those ELPs from the library predicted to be thermoresponsive, revealing several handles to tune their mechanical and structural properties: chain hydrophilicity/hydrophobicity or block distribution control the viscoelasticity and thermoresponsiveness, whereas ELP concentration defines the network permeability. Our findings provide an avenue to accelerate the design of *de novo* ELPs with bespoke phase behavior and material properties.

## Introduction

Many natural materials like bone, silk cocoons, mussel threads, or insect wings consist of a self-assembling biopolymeric scaffold formed by structural proteins such as collagen, elastin, silk fibroin, or resilin.^1^ These structural proteins display impressive mechanical, structural, and biological traits from a materials science perspective, such as the ability to combine toughness and strength in a lightweight material, to interact with the environment or to promote cell adhesion.^2,3^ Those traits arise from the collective effects of nanoscale features encoded in the amino acid sequence of structural proteins, such as the frequency and distribution of ordered/disordered, hydrophobic/hydrophilic, charged/uncharged, or chemical/physical cross-linking blocks.^4^

Structural proteins have inspired material scientists to manufacture multifunctional materials for fields including medicine,^5^ energy harvesting,^6^ and biosensing.^7^ However, harvesting structural proteins from nature is inefficient and constrains us to using only biopolymers selected for by evolution.^8^ Luckily, developments in bioprocess engineering and molecular and synthetic biology enable the use of microbial cultures for the biofabrication of *de novo* structural proteins.^3,9^ This allows us to rationally design new protein sequences that encode well-defined chain topologies and interactions to control the mechanical, structural, and biological features of these proteins.

Elastin is one of the most studied structural proteins.^10^ It is a key component of the extracellular matrix of mammals, conferring elasticity to tissues like blood vessels, skin, and lungs.^11^ Its precursor, tropoelastin, is rich in hydrophobic VPGVG blocks and displays a lower critical solution temperature (LCST) phase behavior: it is water-soluble below a transition temperature (*T*_t_), but it coacervates and phase-separates above it.^12^ This has inspired the development of elastin-like polypeptides (ELPs)^13^ and derivatives thereof for the manufacture of dynamic and thermoresponsive materials in fields like tissue engineering,^14^ drug delivery,^15^ microfluidics,^16^ and actuation.^17^ ELPs have been used also for the development of micelles, vesicles, artificial cells, membraneless organelles, and as models of intrinsically disordered proteins.^13,18−25^ Like tropoelastin, ELP solutions also display LCST phase behavior.^26,27^ ELPs are intrinsically disordered and highly dynamic both below and above the *T*_t_.^28,29^ Above the *T*_t_, ELP–ELP interactions become favored over ELP–solvent interactions, and thus the solution segregates into ELP-rich and solvent-rich phases.^29^ The thermoresponsiveness of ELPs can be controlled by sequence design (e.g., block arrangement or molecular weight) or by processing conditions (e.g., protein concentration or solution conditions),^30^ and numerous studies have shown the ability of ELPs to form a rich landscape of sophisticated nanostructures above the *T*_t_.^31−33^

ELPs typically consist of repetitions of the pentapeptide building block VPGXG,^26,27^ where X is referred to as the guest amino acid and can be any amino acid except proline.^13^*De novo* ELPs are designed following a modular approach, in which variations of the VPGXG building block are encoded into a polypeptide chain that is produced via microbial fermentation. Early research focused on elucidating the molecular origins of the elasticity and LCST phase behavior of ELPs^27,34−36^ and on characterizing selected ELP for the manufacture of films,^37^ fibers,^31^ nanoparticles,^38^ or hydrogels.^39^ This unveiled the influence of parameters like guest amino acid^40^ or polypeptide molecular weight (MW).^41^ Yet, precise heuristics that link sequence design to macroscopic dynamic, mechanical or structural features for *de novo* ELPs remain largely unknown. It was only recently that some studies started uncovering such sequence–property relationships.^42−45^

The presence of a *T*_t_ at physiological temperature is a requirement for ELPs in several biomedical applications, but the value of the *T*_t_ is usually unknown for *de novo* ELP sequences. To that end, molecular dynamics (MD) simulations can aid to predict the LCST phase behavior of *de novo* ELPs by rapidly and simultaneously screening multiple permutations of building blocks^46^ before synthesizing selected ELPs^28,34,36,43^. However, the predictive power of MD simulations for the design *de novo* ELPs is yet to be fully harnessed. It is generally stated that simulation times in the microsecond range^28^ or advanced sampling methods like replica-exchange^43^ are needed to attain statistically converged sampling of ELPs via MD simulations. Furthermore, the lack of a standardized framework to interpret the outcome of MD simulations has hindered their use for the design of *de novo* ELPs. However, several studies have claimed that MD simulations in the 10–100 ns range can capture the LCST phase behavior of different homopolymeric or multiblock ELPs. This was achieved by monitoring molecular properties like radius of gyration, intra-ELP hydrogen bonds, secondary structure, or water molecules in the hydration layer of ELPs^28,34,36,43,47−49^ and linking them with the molecular collapse typically associated with the LCST phase behavior of ELPs.^43,50^

Here we propose a different approach to develop a predictive computational framework for the LCST phase behavior of *de novo* ELPs. Our work draws inspiration from quantitative structure–activity relationship (QSAR) and quantitative structure–property relationship (QSPR) approaches^51,52^ and uses relatively short (150 ns per replica), fully atomistic MD simulations. We validated the model by synthesizing a library of eight *de novo* ELPs, which sweeps varying numbers and locations of hydrophobic/hydrophilic and physical/chemical cross-linking blocks. Two clusters were identified among our library based on principal component analysis (PCA) of a set of 27 molecular properties sampled from MD simulations. These clusters, but not the individual molecular properties, coincided with the presence or absence of LCST phase behavior below 37 °C, as determined experimentally. Subsequently, we developed a regression model that accurately discriminated *de novo* ELPs according to their phase behavior based on data from MD simulations. Finally, we used experimental tools to investigate properties inaccessible to current computational methods, but that are critical for the development of ELP-based materials. These include rheological properties, secondary structure and supramolecular microstructure of the ELP library. Together, this approach allowed us to bridge sequence-level features with nano-, micro-, and macroscopic structural and mechanical properties, providing an avenue for the rational design of *de novo* ELP-based materials with bespoke properties.

## Materials and Methods

### Synthesis and Characterization of the ELP Library

Synthetic DNA sequences encoding for the full length of the different elastin-like polypeptides (ELPs; Table 1) were purchased from GeneArt (Regensburg, Germany). The DNA fragments were then transformed into an electrocompetent *E. coli* K12 strain proprietary of DSM (Delft, The Netherlands). The correct transformation of plasmids containing the ELP genes was confirmed by agarose gel electrophoresis. To that end, plasmids were isolated using the NucleoSpin plasmid DNA purification kit (Macherey-Nagel) according to the manufacturer’s protocol. Transformants were randomly selected and used for bacterial fermentation in 2 L shake flasks containing 500 mL of Terrific Broth medium. Cultivation was performed at 27 °C and ELP expression was induced with l-arabinose when the optical density of the culture at 600 nm reached 0.6. After overnight expression, cells were harvested by centrifugation at 5500 rcf for 20 min at 4 °C. The supernatant was decanted, and the cell pellets were subjected to a freeze–thaw cycle to rupture them. Thereafter, cell pellets were resuspended in PBS and tip sonicated to enhance the release of ELP. The LCST phase behavior of ELPs allowed for their purification via inverse temperature cycling.^53,54^ The aggregation of ELPs was triggered by adding 2 M NaCl and incubating the solutions at 42 °C for 1 h. For ELPs containing glutamic acid, the pH was adjusted to 4 to protonate glutamic acid residues and facilitate the coacervation.^55^ Purified ELPs were desalted in 3000 MWCO Amicon ultra-15 centrifugal filter units (MilliporeSigma). The desalted materials were then resuspended in Milli-Q water, flash-frozen, and lyophilized, followed by storage at −20 °C until further use. The purity of ELPs was assessed via sodium dodecyl sulfate–polyacrylamide gel electrophoresis (SDS-PAGE) using NuPAGE 4–12% Bis-tris gels (Invitrogen). Mark12 unstained standard (Thermo Fisher) was used as protein ladder. Gels were stained using SYPRO Red gel staining agent (Invitrogen) following the manufacturer’s protocol. Total amino acid analysis was performed using the Accq Tag method after chemical hydrolysis (Waters). The theoretical hydrophobicity of the ELPs was calculated using the Kyte–Doolittle scale.^56^ Their molecular weight was assessed via intact LC-MS.

**Table 1:** Name, Amino Acid Sequence, and Theoretical and Real (Determined via LC-MS) MW of the ELP Library

name	sequence	theoretical MW (kDa)	experimental MW^a^ (kDa)
E_AE_	[(IPAVG)(VPGVG)_2_(VPGEG)(VPGVG)_2_(IPAVG)]_12_	35.6	35.4
E_AE,triblock_	(IPAVG)_12_[(VPGVG)_2_(VPGEG)(VPGVG)_2_]_12_(IPAVG)_12_	35.6	35.4
SE_E_	[(GAGAGS)(VPGVG)_2_(VPGEG)(VPGVG)_2_(GAGAGS)]_12_	34.7	34.6
E_KE_	(VPGKG)_12_[(VPGVG)_2_(VPGEG)(VPGVG)_2_]_12_(VPGKG)_12_	35.6	35.5
E_AI_	[(IPAVG)(VPGVG)_2_(VPGIG)(VPGVG)_2_(IPAVG)]_12_	35.6	35.3
E_AI,triblock_	(IPAVG)_12_[(VPGVG)_2_(VPGIG)(VPGVG)_2_]_12_(IPAVG)_12_	35.4	35.3
SE_I_	[(GAGAGS)(VPGVG)_2_(VPGIG)(VPGVG)_2_(GAGAGS)]_12_	34.5	34.4
E_KI_	(VPGKG)_12_[(VPGVG)_2_(VPGIG)(VPGVG)_2_]_12_(VPGKG)_12_	35.4	35.3

a Note: the error associated with this measurement is 0.5–1.0 Da.

### Computational Modeling

Input structures for molecular dynamics (MD) simulation were created based on the ELP sequences from Table 1. To reduce the computational cost of these simulations, we used 5-mer sequences instead of the 12-mer used experimentally (Table S1). Extended conformations of each ELP were built using the software Avogadro (version 1.2.0).^57^ MD simulations were performed using the software NAMD 2.13 developed by the Theoretical and Computational Biophysics Group in the Beckman Institute for Advanced Science and Technology at the University of Illinois at Urbana–Champaign.^58^ The Chemistry at HARvard Macromolecular Mechanics (CHARMM36) force field was used for these simulations.^59^ This force field is widely used for studying proteins, including ELPs.^28,43,47^ The atomic structures were visualized using the Visual Molecular Dynamics (VMD) graphics software.^60^

#### Implicit Solvent Simulations

Each extended ELP structure was subjected to MD simulations in implicit solvent to obtain a folded structure. The equilibration and folding of ELPs was assessed by the evolution of the root mean squared displacement (RMSD) of their atomic positions. First, the structure was subjected to energy minimization for 20000 time steps to relax the polypeptide, using the steepest descent algorithm. This was followed by Langevin dynamics in Generalized Born implicit solvent.^61^ A simulation step of 2 fs was applied at 310 K for a total simulation time of 50 ns. The short-range electrostatic interactions and Lennard-Jones interactions were evaluated with a cutoff of 18 Å and a switch distance of 16 Å.^62^

#### Explicit Solvent Simulations

Simulations in explicit water were performed at 310 K. The final structures obtained in implicit solvent simulations were solvated in a TIP3P water box with 3D periodic boundary conditions. The distance between any ELP atom and the edge of the periodic box was at least 12 Å to avoid spurious effects of self-interactions for the ELP chain. Na^+^ and Cl^–^ ions were added to neutralize the charge of the system in simulations containing ionic ELPs. The ShakeH algorithm was applied to all the bonds containing hydrogen atoms. The energy of the systems was minimized for 20000 timesteps, followed by Langevin dynamics for 150 ns using the NPT ensemble at 310 K and pressure (1 bar) was exerted through the Nosé–Hoover Langevin piston. The long-range electrostatic Coulombic interactions were calculated using particle mesh Ewald method with a grid spacing of 1 Å. A cutoff distance of 12 Å was applied for electrostatic and van der Waals interactions, with a switch distance of 10 Å to avoid hard cuts.^62^ A time step of 2 fs was applied. The backbone of the ELPs was restrained for the first 1 ns of simulation. Three replicas were performed for each system, and simulation data was sampled every 0.2 ns.

#### Data Analysis

Molecular properties were sampled from the last 50 ns of the MD trajectories. The evolution of the RMSD of the atomic positions during the simulation was analyzed for the protein backbone using the RMSD Trajectory Tool from VMD. The count of intraprotein and protein–solvent hydrogen bonds throughout the simulation trajectories was analyzed using the Hbonds plugin from VMD, using a distance cutoff of 3.5 Å and a D–H–A angle cutoff of 30° as geometric criteria. Custom TCL scripts were developed to analyze in VMD the solvent accessible surface area (SASA; using a standard water probe radius of 1.4 Å), the radius of gyration of ELPs, and the evolution of the secondary structure (using the STRIDE algorithm).^63^ Water molecules in the hydration shell of the ELP were defined as those within 3.15 Å from the ELP backbone. The interaction energies were calculated using the molecular mechanics energy function in NAMD 2.13.

For exploratory data analysis purposes, principal component analysis (PCA) was used. PCA searches for the variable subspace summarizing the main features of the data.^64^ PCA transforms the original (correlated) variables (in this case, the molecular properties sampled from MD simulations) into a lower number of uncorrelated variables or principal components (PCs). The PCA model equation is
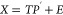
1where *X* is the data matrix (having observations by rows and variables by columns), *T* is the score matrix containing the PCs, *P* is the loading matrix containing the linear combination of the original variables to build the PCs (being *P*′ its transposed), and *E* is the residual matrix.

A predictive model for MD data was developed via a supervised model based on partial least-squares regression discriminant analysis (PLS-DA). PLS is a multivariate statistics method commonly used to predict an output variable *y* from a set of *X* predictors.^65^ PLS, similarly to PCA, reduces the dimensionality of the *X* variable space by finding the linear combinations of predictors that best summarize the *X* variable space and best predict the output variable. Therefore, the components found by the PLS algorithm maximize the covariance between *X* and *y*. The scores (or PLS components) are obtained as
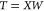
2where *W* is the normalized weights matrix. On one hand, these scores reconstruct well the original predictor matrix *X* = *TP*′ + *E*. On the other hand, *y* can be predicted using the score matrix^66^ as
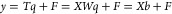
3where *F* is the residual matrix and *b* are the PLS coefficients. In our case, the response variable is not quantitative but qualitative (presence or absence of LCST phase behavior), and therefore the discriminant version of PLS (PLS-DA) was used.^67^ To perform PLS-DA, the presence or absence of LCST phase behavior below 37 °C was coded as 1 or 0, respectively. When using the regression model to predict, a quantitative value is produced, which is later converted to a qualitative 0/1 taking as threshold the mean between those two values as reference.^67^

Thorough validation of PLS-DA models is needed when dealing with large data sets. Single cross validation approaches, where the model is built with a subset of samples and validated with an external set, often leads to too optimistic results.^68,69^ That is why a more stringent double cross validation procedure was used here, which comprised the following steps:

1. The 8 MD simulations (one per ELP, including three replicas for each ELP) were split into two groups: training (including simulations 1–7) and test (including simulation 8).
2. The simulations in the training set were further split into two groups: model (including simulations 1–6) and validation (including simulation 7).
3. The six model simulations were used to build a partial least-squares regression discriminant analysis model (PLS-DA).
4. The validation simulation was then projected into the PLS-DA model. The number of components of the PLS-DA model was selected at this step, including the minimum number of components needed to obtain perfect classification for each ELP (presence/absence of LCST phase behavior below 37 °C) of the validation set.
5. The PLS-DA model built in step 3 with as many components as defined in step 4 was used to predict the result of the test set, left out in step 1.

Steps 2–5 were afterward repeated six times, switching the validation simulation from 1 to 6, and using the remaining ones to build the model. In the same way, steps 1–5 were then repeated, switching the test simulation from 1–7, using the remaining ones to build the model and validate it.

### Hydrogel Formation and Characterization

Experiments were performed using ELP solutions in Milli-Q water prepared at 4 °C and with a polypeptide concentration of 15 wt %. This concentration has been reported to yield stiff ELP hydrogels at 37 °C.^70^ In all cases (except for chemical hydrogels) solutions were incubated at 4 °C overnight. For the lysine-containing ELPs, chemical hydrogels were formed by dissolving the polypeptides in Milli-Q water containing an excess of glutaraldehyde as cross-linking agent (40:1 glutaraldehyde:ELP molar ratio). The dissolution in glutaraldehyde solutions of lysine-containing ELPs was performed on ice for 10 min to facilitate ELP dissolution. Higher temperatures were avoided during this step, as they accelerated the reaction between ELP and glutaraldehyde and rapidly formed a stiff chemical hydrogel that could not be pipetted onto the rheometer.

#### Turbidimetry

The ability of ELPs to display LCST phase behavior below 37 °C was analyzed via optical density measurements using a microplate reader (Multiskan GO, Thermo Scientific). 100 μL of each ELP solution (15 wt %) were loaded in a 96-well plate at 4 °C. The plate was then introduced in the microplate reader preheated at 37 °C, and the absorbance of the solutions was recorded at 350 nm.

#### Rheology

Rheological characterization was performed to assess the ability of ELP solutions to form hydrogels, and to characterize their viscoelastic properties. Experiments were performed using small amplitude oscillatory shear rheology on a stress-controlled rheometer (Anton Paar MCR 501) equipped with a cone–plate geometry with a diameter of 20 mm and a cone angle of 1°. The temperature was controlled by a Peltier system. For each test, 42 μL of the ELP solutions (15 wt %) were loaded at 4 °C onto the bottom plate using a pipette, followed by equilibration for 5 min. Solutions without glutaraldehyde were incubated overnight at 4 °C before loading them onto the rheometer. For experiments with glutaraldehyde, ELPs were dissolved in a Milli-Q water-glutaraldehyde solution for 10 min on ice, and quickly pipetted onto the Peltier plate. Low viscosity mineral oil (Sigma-Aldrich) was applied to the air-sample interface around the measuring geometry to prevent water evaporation. The *T*_t_ of ELP solutions was analyzed with a temperature sweep between 4 and 37 °C (with a heating rate of 1 °C/min). The reversibility of gelation was assessed with three temperature cycles between 4 and 37 °C (with a heating/cooling rate of 1 °C/min). Holding times of 30 min were applied at 4 and 37 °C, after each temperature ramp. Strain sweeps were performed from 0.01% to 15% at a frequency of *f* = 1 Hz to evaluate the linear viscoelastic region of these hydrogels. Frequency sweeps were carried out at 37 °C from 0.01 to 15 Hz, using a constant strain amplitude γ = 0.3%.

#### Fourier Transform Infrared Spectroscopy (FTIR)

Freeze-dried samples of ELP solutions and hydrogels were analyzed by FTIR to assess their secondary structure below and above *T*_t_, respectively. A total of 80 μL of cold ELP solutions were placed in microcentrifuge tubes. Samples before gelation were directly submerged in liquid N_2_ for 1 min. Hydrogel samples were prepared by incubating ELP solutions at 37 °C for 1 h (physical hydrogels) or 2 h (chemical hydrogels) before flash-freezing them in liquid N_2_ for 1 min. All samples were then lyophilized and cryo-fractured prior to FTIR analysis. Infrared spectra were measured in a Bruker Vertex 70 Attenuated Total Reflectance FTIR device equipped with a Harrick split pea accessory. For each measurement, 64 scans with a resolution of 2 cm^–1^ were recorded in the range of 650 to 4000 cm^–1^. The secondary structure of the polypeptides is related to the C=O stretching vibration and can be determined by performing peak deconvolution over the amide I region (1595–1705 cm^–1^). This was done using the lmfit package for curve fitting from Python. The deconvolution was carried out using five primary peaks assigned to different secondary structures: 1620 cm^–1^ (β-sheet), 1645, 1660, and 1670 cm^–1^ (random coil/helix), and 1700 cm^–1^ (β-turn).^71^ The peak positions were allowed to shift 4 cm^–1^ to obtain a reconstituted curve as close as possible to the original spectra. The amide I region from all spectra was normalized to its highest value, to facilitate the comparison between different samples. We used the Levenberg–Marquardt least-squares method, and a Gaussian model was selected for the band shape.

#### Mesh Size Determination

We estimated the typical mesh size ξ (distance between physical or chemical cross-linking points) of the ELP hydrogels from the measured *G*′, based on rubber elasticity theory:^72^
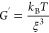
4Equation 4 can be applied to gels and physically cross-linked networks of flexible chains,^72^ with *k*_B_*T* representing the thermal energy at the temperature *T* used for *G*′ determination.

#### Fluorescent Recovery after Photobleaching (FRAP)

Diffusion coefficients of fluorescent dextran probes in ELP hydrogels (15 wt %) were determined via FRAP experiments. ELPs were dissolved in fluorescein isothiocyanate (FITC)-labeled dextran solutions in Milli-Q water (40 and 150 kDa, 1 mg/mL, Sigma) at 4 °C. FRAP experiments were performed on a Leica Stellaris 8 Falcon confocal microscope at 37 °C equipped with a white light laser. The FRAP protocol started with 10 images at low laser power and a pixel dwell time of 3.6125 μs to determine the baseline fluorescence. Subsequently, a square region of 37.5 × 37.5 μm was photobleached using an excitation wavelength of 491 nm at 100% intensity for 7 s. A time series was then recorded to follow fluorescence recovery, acquiring a frame (pixel dwell time of 3.6125 μs, image size 512 × 128) every 0.33 s for the first 20 s, and every 1 s thereafter. FRAP data was subjected to full-scale normalization,^73^ using the fluorescence of the whole imaging area to normalize and correct for laser fluctuations, photobleaching during image acquisition, and fluorescence loss during photobleaching. The fluorescence recovery was analyzed by fitting the normalized data to a single exponential curve using the curve_fit function of the SciPy module from Python:
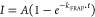
5where *A* corresponds to the plateau intensity after fluorescence recovery, *k*_FRAP_ is the recovery rate, and *t* is the time after photobleaching. We calculated the free diffusivity *D*_0_ of pure dextran solutions using the Stokes–Einstein relationship:
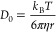
6where *k*_B_*T* is the thermal energy at the temperature *T* used in FRAP experiments (37 °C), η is the viscosity of the solvent (0.692 mPa.s for Milli-Q water at 37 °C), and *r* is the dextran particle radius (4.5 nm for 40 kDa dextrans, 8.5 nm for 150 kDa dextrans). To calculate the diffusivity *D*_hydrogel_ of dextrans within ELP hydrogels, we assumed that *D* ∼ *k*_FRAP_·*L*^2^, where *L* is the characteristic length scale of our FRAP experiments (the size of the bleached area, 37.5 μm). Given that the size of the bleached spots was constant in our FRAP experiments, it follows that
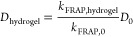
7

#### Scanning Electron Microscopy (SEM)

Freeze-dried samples of ELP hydrogels were analyzed by SEM to investigate their microstructure. A total of 80 μL of each solution (15 wt %) was incubated in microcentrifuge tubes at 37 °C for 1 h (physical hydrogels) or 2 h (chemical hydrogels). The tubes were then submerged in liquid N_2_ for 1 min, followed by lyophilization. The resulting samples were then cryo-fractured and coated with a 2 nm layer of iridium using a Q150T S/E/ES sputter coater (Quorum Technologies). SEM images were obtained using a FEI Teneo LoVa microscope with trinity detector. Analysis of the pore sizes of SEM samples was performed using the software ImageJ^74^ by measuring the diameter of 30 randomly selected pores.

## Results and Discussion

### Predicting the Phase Behavior of an ELP Library

**Figure 1:**
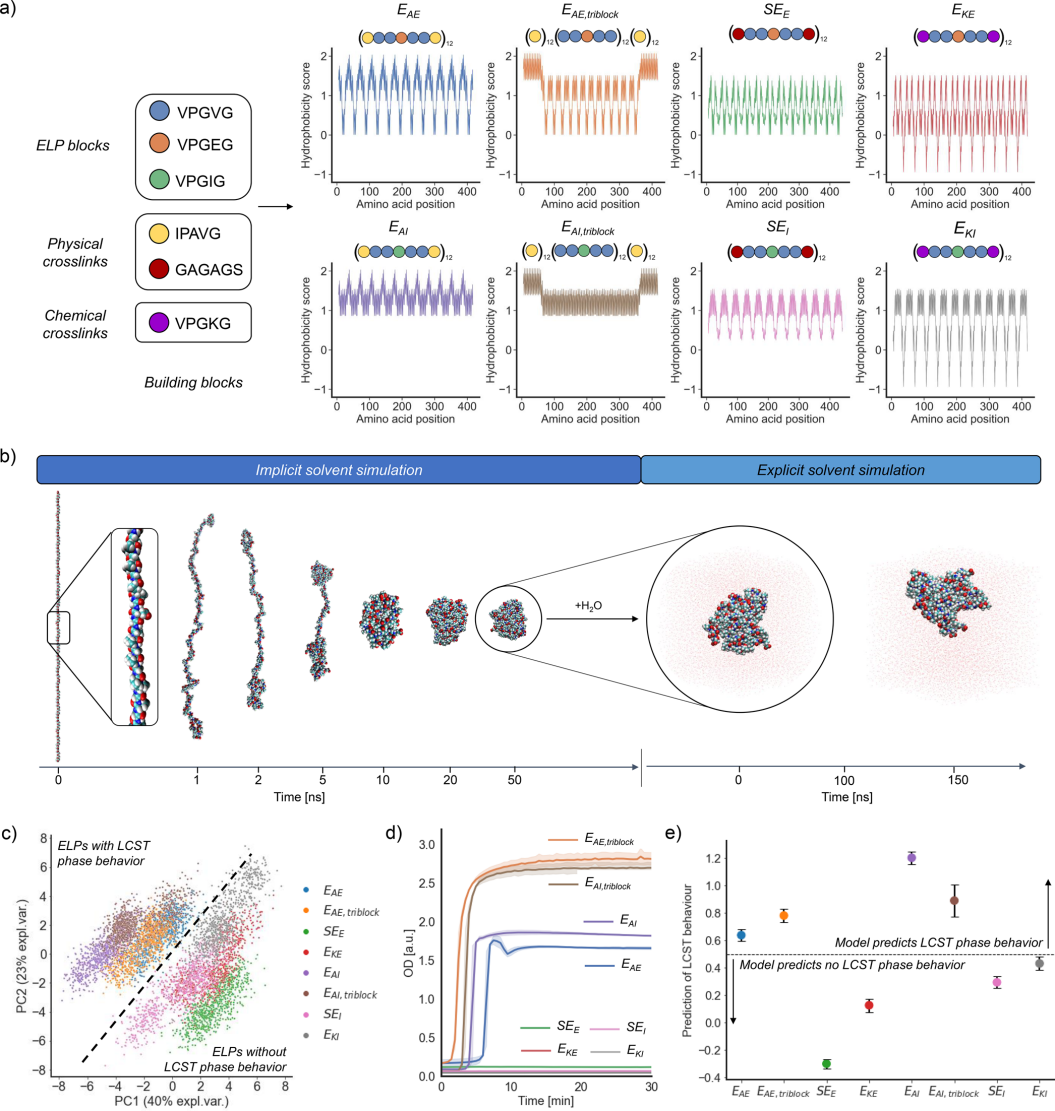
ELP library design, MD simulations and experimental validation of LCST phase behavior. (a) Schematic representation of distribution of building blocks along the sequences of our ELP library, together with their hydropathy plots as calculated using the Kyte-Doolittle scale. (b) Screenshots of the folding process (in implicit solvent) and dynamic run (in explicit solvent) of MD simulations for E_AE,triblock_. (c) Principal component analysis (PCA) scores plot. Each dot represents a time point from the last 50 ns of MD simulations (*n* = 3) on principal components 1 (*x*-axis) and 2 (*y*-axis). A dashed line separates the two clusters detected. (d) Time evolution of the optical density measured for ELP solutions in Milli-Q water (15 wt %) at 37 °C (*n* = 2). (e) Predictions of the LCST phase behavior of our ELP library at 37 °C with a partial least-squares regression discriminant analysis (PLS-DA) model that uses molecular properties sampled from MD simulations and optical density measurements as input data. The value on the *y*-axis indicates the prediction of the LCST phase behavior for each ELP. When the prediction is above 0.5 (dotted line), the model predicts the presence of LCST phase behavior. The error bars represent the 99% confidence intervals from the predictions per ELP during the double cross-validation procedure.

A library of eight *de novo* ELPs with size-matched molecular weights (MWs) of 34.5–35.6 kDa was designed (Table 1). All ELPs contained 48 repeats of the VPGVG building block, while the following molecular modulators were varied: guest amino acid, location of the cross-linking blocks, and identity of the cross-linking block. For a given MW, the *T*_t_ can be tuned by changing the amino acid in the X position.^27,40^ We hence compared a nonpolar guest amino acid (isoleucine, I) versus an ionic one (glutamic acid, E). To promote the formation of a bicontinuous hydrogel network, we used physical (IPAVG, GAGAGS) or chemical (VPGKG) cross-linking blocks. IPAVG blocks in E_AE_, E_AI_, E_AE,triblock_, and E_AI,triblock_ can form kinetically arrested gels.^70^ GAGAGS blocks in SE_E_ and SE_I_ were based on β-sheet-forming blocks from silk fibroin. These blocks can arrest ELP phase separation by forming β-sheet structures.^39,45^ Finally, VPGKG blocks were introduced in E_KE_ and E_KI_ to form covalent bonds between ELP chains via lysine (K) residues upon addition of glutaraldehyde.^32^ To test how the positioning of cross-linking blocks affects the LCST phase behavior and hydrogel functionality, we investigated multiblock (E_AE_ and E_AI_) and triblock arrangements (E_AE,triblock_ and E_AI,triblock_). The effect of these modulators can be visualized by the Kyte–Doolittle hydropathy scale^56^ of these ELPs (Figure 1a).

Previous research suggested that ELPs with as few as 10 VPGXG blocks manifested LCST phase behavior in MD simulations.^49^ Therefore, to reduce the computational cost of our MD simulations, we simulated shorter multimers of our library, equivalent to ELPs with 35 VPGXG blocks (Table S1). Implicit solvent simulations were first run, starting with the fully extended ELP chain, to achieve a folded structure (Figure 1b). The resulting structure was then solvated in explicit water and, when needed, its electrical charges were adjusted to achieve charge neutrality. The solvated system was then run in triplicate for 150 ns more for each ELP. After 100 ns, ELPs were stable in terms of root-mean-square deviation (RMSD) of atomic positions (Figure S1–S2), indicating that the system was sufficiently equilibrated to begin data sampling. Thereafter, we sampled 27 different molecular properties every 0.2 ns (equivalent to 100000 timesteps) for the next 50 ns. These molecular properties were chosen based on prior studies showing their connection with LCST phase behavior for specific ELP sequences^28,43,47,49^. This yielded 250 data points per molecular property and per replica. These properties include intrapeptide hydrogen bonding, radius of gyration, RMSD, solvent accessible surface area (SASA), secondary structure, and water molecules in the hydration layer, among others. A full description of all the molecular properties sampled can be found in Table S2.

Individually, these molecular properties revealed mostly small differences among the different ELPs in our library (Figures S3–S6). Inspired by QSAR and QSPR approaches,^51,52^ where a set of descriptors (the molecular properties obtained from MD simulations) are connected with a property (presence or absence of LCST phase behavior), we decided to analyze these molecular properties collectively, exploring their multidimensional variable space via PCA. By analyzing all data points, rather than averaging them, we increased the statistical accuracy of our study, while accounting for temporal fluctuations in the molecular properties during the simulation. PCA summarizes the main features of a high-dimensional multivariate data set (the output of our simulations) into a few artificial variables, called principal components (PCs). We found that the first two PCs accounted for 63% of the variance observed in the 27-dimensional data set. As shown in Figure 1c, where each dot corresponds to a time point in the MD simulations, the first two PCs divided our ELPs into two distinct clusters: E_AE_, E_AE,triblock_, E_AI_, and E_AI,triblock_ (all of which contain IPAVG blocks) on the one hand, and SE_E_, E_KE_, SE_I_, and E_KI_ (which contain GAGAGS or VPGKG blocks) on the other hand.

We then explored the connection between the PCA clusters and the experimental phase behavior of our library at 37 °C. ELPs were heterologously expressed in *E. coli*. Purification was performed by inverse temperature cycling.^55^ The correct transformation of plasmids containing the ELP genes and the purity of the recovered ELPs were confirmed by agarose (Figure S7) and sodium dodecyl sulfate polyacrylamide gel electrophoresis (Figure S8), respectively. The resulting ELPs were within 2% of the expected amino acid composition, as determined by total amino acid analysis (Table S3). The correspondence between the theoretical and experimental MW was confirmed by intact LC-MS (Table 1). LCST phase behavior is typically accompanied by a turbidity change for ELP solutions.^36^ Thus, we prepared cold (4 °C) 15 wt % ELP solutions in Milli-Q water and monitored their optical density when placed in a microplate reader at 37 °C (Figure 1d). Interestingly, ELPs divided into the same clusters shown by PCA: E_AE,_ E_AI_, E_AE,triblock_, and E_AI,triblock_ polypeptides showed an increased turbidity (indicative of an LCST phase behavior), whereas SE_E_, E_KE_, SE_I_, and E_KI_ polypeptides did not. Apparently, the collective evaluation via PCA of multiple molecular properties obtained from short MD simulations (150 ns) was a suitable tool to classify the LCST phase behavior of ELPs.

However, PCA provides an unsupervised exploratory model but does not make any predictions about the significance of the two clusters identified. To speed up the development of *de novo* ELPs, we also need to be able to predict the LCST phase behavior of ELPs not used to build the model. To address this, we switched to a supervised model. This model also included experimental data from optical density measurements: the presence or absence of LCST phase behavior below 37 °C was coded in the model as 1 and 0, respectively. Our objective was to identify the combination of MD molecular properties in our data set that discriminated ELPs based on their LCST phase behavior below 37 °C. We did so by applying a partial least-squares regression discriminant analysis (PLS-DA) model.^66,67^ PLS-DA is a suitable approach for the analysis of a high-dimensional data set (in this case, our 27 molecular properties) with a binary outcome (presence or absence of LCST phase behavior). The principle behind PLS-DA methods is similar to PCA, but maximizing covariance between the data set of molecular properties and their phase behavior below 37 °C, instead of maximizing the variance observed within the molecular properties only. This generated a model that summarized both the molecular properties obtained via MD simulations and predicted the outcome (presence or absence of LCST phase behavior below 37 °C).

To develop our PLS-DA model, we applied a rigorous double cross validation procedure in an iterative manner (see Experimental section) so that the PLS-DA coefficients for the MD molecular properties were predicted 7 times for each ELP and then averaged. When an ELP simulation was left out of the PLS-DA model and then predicted, a numeric value was produced (Figure 1e). When that value was above 0.5,^67^ the model predicted that the ELP displayed LCST phase behavior below 37 °C. The predictions for each simulation were combined in 99% confidence intervals. When the confidence intervals do not cross the boundaries of the correct class (presence/absence of LCST phase behavior below 37 °C), the experiment is properly predicted. This approach mimics real-world design-build-test-learn cycles,^75^ where a model is first built with the current knowledge, then a new experiment is designed using that knowledge and predicted through the model, and finally the predictions are validated experimentally. When leaving E_AE_, E_AE,triblock_, E_AI_, and E_AI,triblock_ out of the model and then using the model to predict their LCST phase behavior, the 99% confidence interval was entirely above 0.5 (presence of LCST phase behavior). In turn, the same interval for SE_E_, E_KE_, SE_I_, and E_KI_ was entirely below 0.5 (absence of LCST phase behavior). The regression coefficients of the PLS-DA model for each molecular property showed small error bars and can be found in Figure S9 and Table S4. Thus, the PLS-DA model agreed with the PCA and turbidity data, attaining a 100% prediction accuracy. The data indicated that the most influential molecular properties for the LCST phase behavior were hydrophobic or hydrophilic SASA, number of water molecules in the hydration layer of an ELP, number of hydrogen bonds between the ELP and its hydration layer, β-sheet content, RMSD of the polypeptide, and electrostatic energy.

To sum up, previous MD studies of ELPs typically assessed molecular properties individually or in pairs, which helped to establish the links between some molecular properties (e.g., hydrophobic or hydrophilic SASA) and LCST phase behavior. However, this usually required long simulation times or computationally expensive advanced sampling methods,^28,43^ and the reported results were typically associated with large uncertainties. Instead, here we achieved an unambiguous separation between the ELPs that display LCST phase behavior below 37 °C and those that do not by collectively analyzing 27 molecular properties (via PCA and PLS-DA) using relatively short simulation times in explicit solvent (150 ns). This finding hints that MD simulations hold great potential to rapidly screen and predict qualitative trends in the phase behavior of ELPs.

### Rheological Characterization of the ELP Library

**Figure 2:**
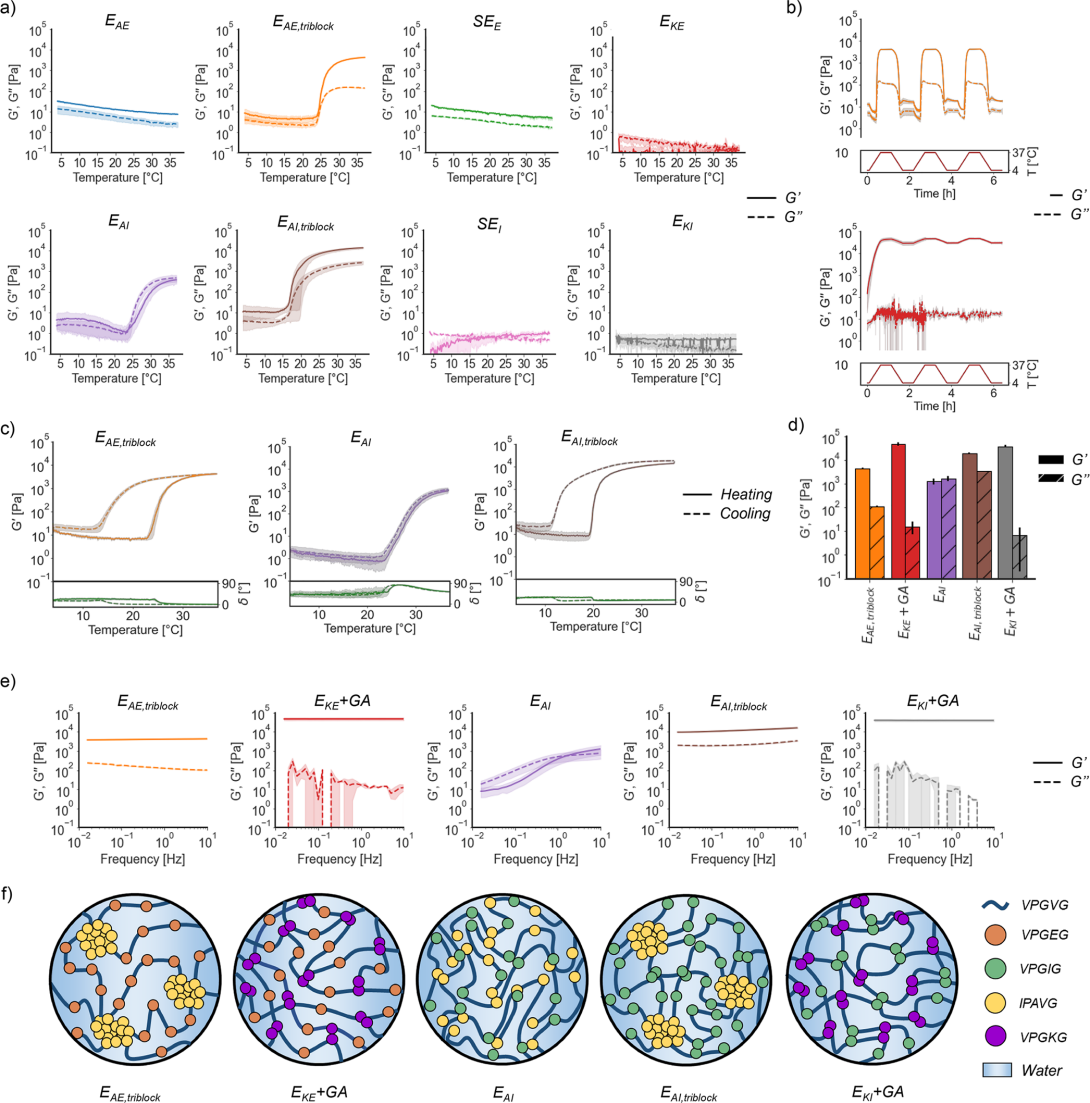
Rheological properties of the ELP library. (a) Rheological characterization (storage modulus *G*′ and loss modulus *G*′′) of ELP solutions in Milli-Q water (15 wt %) during a temperature sweep between 4 and 37 °C (*n* = 4). (b) Thermal cycling of ELP solutions (15 wt %) at a heating/cooling rate of 1 °C/min (*f* = 1 Hz, γ = 0.3%), with 30 min of resting time between temperature ramps: representative reversible physical hydrogel from E_AE,triblock_ (top) and irreversible chemical hydrogel from E_KE_ after glutaraldehyde addition (bottom) (*n* = 2). (c) Hysteresis of ELP solutions forming physical hydrogels over three temperature cycles (*n* = 2). (d) Plateau shear moduli, *G*′ and *G*′′, of hydrogels obtained from ELP solutions in Milli-Q water (15 wt %) at 37 °C (*f* = 1 Hz, γ = 0.3%) (*n* = 2). (e) Frequency-dependence of the shear moduli of ELP solutions in Milli-Q water (15 wt %) (*T* = 37 °C, γ = 0.3%) (*n* = 2). (f) Schematic representation of the cross-linking mechanism above the *T*_t_ for the different ELP designs (color-coded blocks on the right).

Our computational work allowed us to predict the presence of LCST phase behavior for our ELP library. The data suggest that hydrophobic IPAVG blocks are instrumental in triggering the T_t_ below 37 °C in our ELP library, even in spite of the ionic guest residues in E_AE_ and E_AE,triblock_ that are normally detrimental for gelation.^27,32^ Further insight into the sequence-property relationships and their impact on the mechanical properties of ELP hydrogels was obtained via rheological measurements. The *T*_t_ of our ELP library was determined as the temperature at which ELP solutions underwent a sharp increase in their storage (*G*′) and loss (*G*′′) moduli during a temperature sweep from 4 to 37 °C (*f* = 1 Hz, γ = 0.3%) (Figure 2a). Such an increase was only observed for E_AI_, E_AE,triblock_, and E_AI,triblock_ (which formed stiff physical hydrogels), but not for E_AE_. This showed that LCST phase behavior, as measured by optical density, does not necessarily indicate the ability of an ELP solution to form a hydrogel network capable of supporting mechanical stress at the ELP concentration used in this study. The formation of chemical hydrogels via interchain covalent bonds was also assessed for E_KE_ and E_KI_ by adding glutaraldehyde (40:1 glutaraldehyde/ELP molar ratio). Without glutaraldehyde, neither of these ELPs gelled. But upon adding glutaraldehyde, both formed a gel (Figure S10), reaching steady state after ∼2 h (Figure S11). Chemical hydrogels at 37 °C lacked the turbidity typically present in ELP solutions above *T*_t_, indicating that their gelation originated from network formation via covalent bonds, rather than from an LCST phase behavior.

The value of the *T*_t_ was affected by the sequence composition and block distribution. E_AI,triblock_ switched at 18 °C from a solution to a stiff hydrogel. A similar transition was observed for E_AE,triblock_, but at a higher temperature (24 °C), due to its ionic guest amino acid (glutamic acid). In contrast, the shear moduli of E_AI_ increased gradually between 25 and 30 °C. We attribute this to the ability of the non-IPAVG regions in E_AI_ to hydrophobically interact with and hinder the self-interactions of short IPAVG blocks. Early research had shown that a multiblock distribution of hydrophobic blocks in ELPs with the same MW raised the *T*_t_.^76^ Here we demonstrate that a multiblock distribution also weakens the mechanical properties of the network.

Sequences with different physical cross-linking blocks (IPAVG vs GAGAGS) but the same block distribution (i.e., E_AE_ vs SE_E_ or E_AI_ vs SE_I_) also showed differences in thermoresponsiveness. While sequences with IPAVG blocks displayed an increased turbidity in the 4–37 °C range (E_AE_ and E_AI_), equivalent sequences with silk-like blocks (SE_E_ and SE_I_) did not. Even at extended incubation times (4 h) at 37 °C, the shear moduli for SE_E_ and SE_I_ solutions still did not increase (Figure S12). This observation indicates that the increased hydrophilicity and lack of thermoresponsiveness of GAGAGS blocks impeded the gelation of SE_E_ and SE_I_. We found the lack of a *T*_t_ below 37 °C for SE_I_ surprising, given the hydrophobicity of its guest amino acid isoleucine. The inclusion of silk-like blocks is a common strategy to form durable hydrogels, due to the ability of silk-like blocks to physically associate into thermodynamically stable β-sheets.^39,45^ However, our computational and experimental data showed that SE_E_ and SE_I_ do not exhibit*T*_t_ below 37 °C. This differs from previous work showing the ability to gel at 37 °C for ELPs with silk-like blocks and similar MW (though with larger elastin/silk ratios than in our study).^45^ A recent study proposed that aggregation of the ELP regions is a necessary first step to bring the silk-like blocks of silk-elastin polypeptides in close proximity to form β-sheets.^45^ Thus, we hypothesize that the length of the ELP regions (5 VPGXG blocks) between GAGAGS blocks in our library was insufficient to trigger their aggregation.

We also assessed the reversibility of the gelation of these ELP solutions over three temperature cycles between 4 and 37 °C, using a resting time of 30 min between each temperature ramp. The networks disassembled upon cooling for the physical hydrogels (E_AI_, E_AE,triblock_, and E_AI,triblock_), but not for the chemical ones (Figure 2b). Noteworthy differences were identified for compositionally identical ELPs but with building blocks scrambled in a different manner. For instance, E_AI_ and E_AI,triblock_ formed physical hydrogels below 37 °C, yet with different hysteresis behavior (Figures 2c and S13). We attribute this behavior to IPAVG blocks, which are known to have hysteresis between solvation and desolvation.^31,32,70^ However, the presence of IPAVG blocks was a necessary condition, but not sufficient, to cause hysteresis: E_AI,triblock_ and E_AE,triblock_ showed hysteresis upon cooling, whereas E_AI_ did not. Multiblock arrangements of IPAVG blocks increased the *T*_t_, prevented hysteresis and led to weaker networks: E_AI,triblock_ networks were one order of magnitude stiffer (*G*′ = 20000 ± 1100 Pa) than those from E_AI_ (*G*′ = 1300 ± 400 Pa) and had a lower *T*_t_, despite having the same number of IPAVG blocks.

The linear viscoelastic (LVE) region of these hydrogels was determined via amplitude sweeps at 37 °C (Figure S14). Triblock designs and chemical hydrogels showed a linear response over the entire range of strain amplitudes (0.01–15%). In contrast, the LVE region for E_AI_ only extended up to strains of ∼3% and the moduli decreased with strain thereafter. We also determined the steady state moduli of these networks after 30 min at 37 °C (*f* = 1 Hz, γ = 0.3%; Figure 2d). For physical hydrogels, *G*′ was 1300 ± 400 Pa (E_AI_), 4500 ± 400 Pa (E_AE,triblock_) and 20000 ± 1100 Pa (E_AI,triblock_), whereas *G*′′ was 1700 ± 400 Pa (E_AI_), 110 ± 10 Pa (E_AE,triblock_), and 3400 ± 200 Pa (E_AI,triblock_). Chemical hydrogels showed little effect of the amino acid sequence, with comparable values for *G*′ (47440 ± 7531 Pa for E_KE_ and 38690 ± 5069 Pa for E_KI_) or *G*′′ (16 ± 9 Pa for E_KE_ and 7 ± 6 Pa for E_KI_). This indicates that the covalently cross-linked macromolecular network has a larger influence on the mechanical properties than the amino acid sequence. Frequency sweeps (Figures 2e and S15) revealed that the relaxation spectra varied across the ELP library. Triblock designs and chemical hydrogels showed an almost frequency-independent behavior for *G*′ and *G*′′ (though the determination of *G*′′ was noisy for chemical hydrogels due to their highly elastic character). By contrast, E_AI_ showed a crossover indicative of stress relaxation at *f* ∼ 2 Hz.

The effect of the hydrophilic/hydrophobic character of the guest amino acid (E vs I) on the viscoelastic properties and *T*_t_ was discerned by comparing E_AE,triblock_ and E_AI,triblock_ hydrogels. The steady state elastic modulus *G*′ for E_AI,triblock_ was 4.6× larger than for E_AE,triblock_, while its loss modulus *G*′′ was 29.5× larger. As mentioned before, the nonpolar midblock of E_AI,triblock_ can likely more easily mix with IPAVG end-blocks. This could promote dissipative interchain hydrophobic contacts. In contrast, the ionic character of VPGEG blocks in E_AE,triblock_ could enhance the aggregation of IPAVG end-blocks, preventing mixing between mid- and end-blocks and reducing the viscous response.

We expected ionic ELPs in our library (E_AE_, E_AE,triblock_, E_KE_, and E_KI_) to form weaker gels or to not gel at all,^43^ because the self-assembly of ionic polymers is typically hindered by electrical repulsion and generally requires that those repulsions are overcome by pH adjustment or by adding counterions.^77^ Indeed, E_KE_ and E_KI_ only gelled after the formation of a macromolecular network upon the addition of glutaraldehyde. In turn, E_AE_ and E_AE,triblock_ did show *T*_t_ in the 4–37 °C range (as shown by turbidity data), but with different mechanical outcomes: E_AE,triblock_ gelled and E_AE_ did not. The already discussed differences between multiblock and triblock IPAVG arrangements were likely exacerbated in E_AE_ and E_AE,triblock_ by the presence of an ionic guest amino acid. It has been proposed that high concentrations of ionic ELPs increase the electrostatic repulsion between chains. This can force some hydrophobic blocks (e.g., IPAVG blocks) to become solvent-exposed, triggering the formation of a hydrogel network.^77^ According to this mechanism, the higher charge density in the midblock of E_AE,triblock_ could facilitate its gelation. The more distributed ionic blocks in E_AE_, together with the reduced length of its IPAVG blocks, would hinder IPAVG–IPAVG associations needed to form the hydrogel network.

Collectively, these observations allowed us to develop an understanding of the mechanisms for network formation in our various ELP solutions that is schematically summarized in Figure 2f. Overall, the highest moduli were obtained for chemical hydrogels. Nonetheless, the viscoelastic properties of physical hydrogels were remarkably high for triblock designs, in the range of tissues like lung, muscle, cartilage or kidney.^78^ This indicates that a rational design of the ELP sequence can deliver materials with mechanical properties in the order of magnitude of chemical hydrogels, but solely relying on physical interactions and without the need of cross-linking agents. This adds to other advantages of physical hydrogels, such as stimuli-responsiveness, self-healing properties, and tailored viscoelasticity.^79^

### Microstructural Characterization of ELP Hydrogels

Our understanding of the nano- and microstructure of ELP hydrogels has been hampered by the intrinsic disorder of ELPs and the high mobility and viscosity of their coacervates.^29^ However, such data is critical for potential applications since it determines the ability of cells to infiltrate hydrogels in tissue regeneration applications and the diffusion of small molecules through the hydrogel network in drug delivery applications. Therefore, we investigated the links between amino acid sequence and the nano- and microstructure of the hydrogel-forming ELPs in our library. We combined Fourier-transform infrared (FTIR) spectroscopy and scanning electron microscopy (SEM) with techniques that probed the hydrogels in a hydrated state. The latter included rheology (discussed in the previous section) and mesh size determination by fluorescence recovery after photobleaching (FRAP).

**Figure 3:**
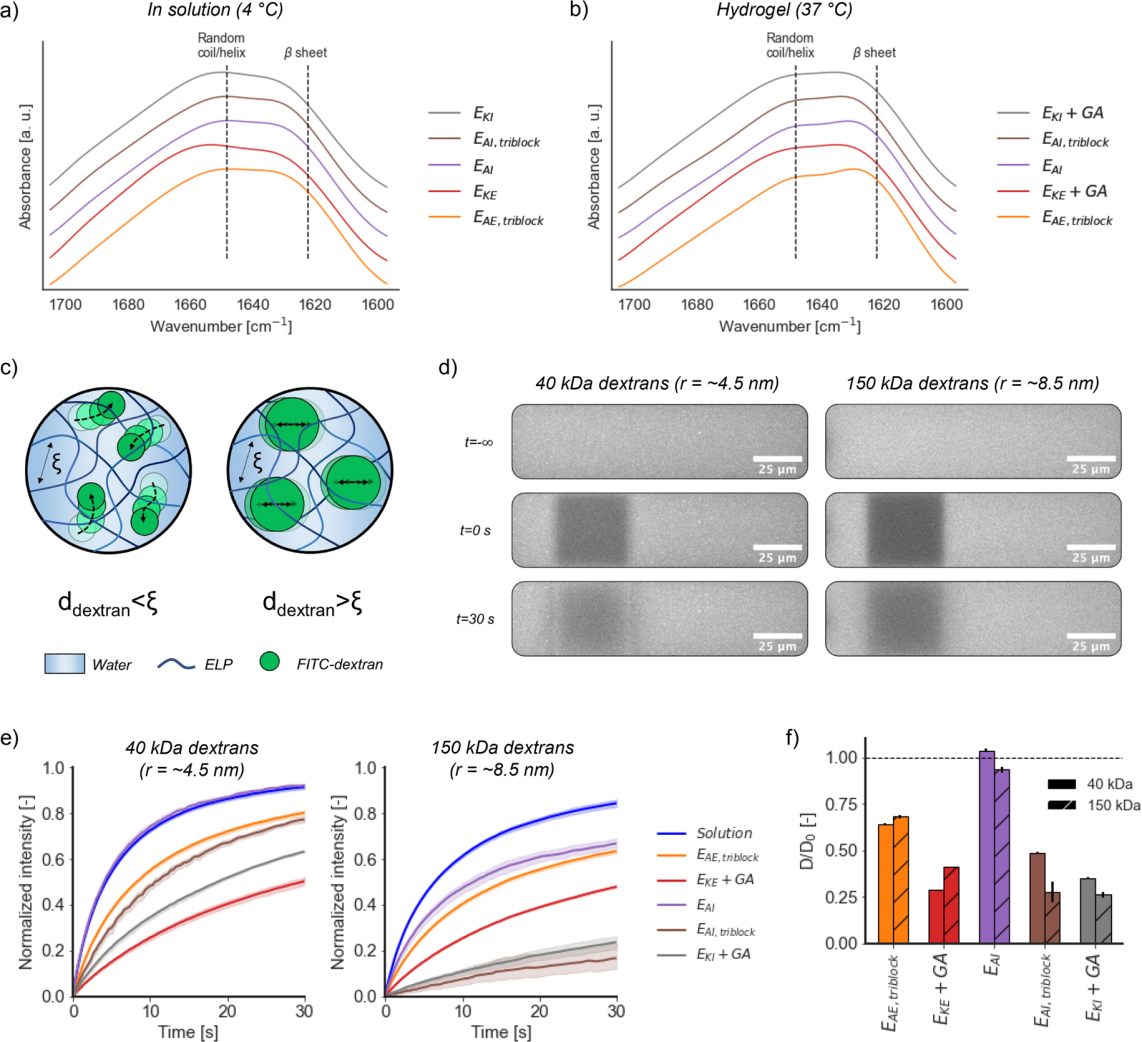
Microstructural characterization of ELP hydrogels. (a) Normalized FTIR spectra of the amide I region for freeze-dried and cryo-fractured ELP samples obtained from 15 wt % solutions after overnight incubation at 4 °C (*n* = 2). Spectra are shifted along the *y*-axis for clarity. (b) FTIR spectra (*n=2*) of the amide I region for freeze-dried and cryo-fractured ELP hydrogels (15 wt %) after incubation at 37 °C for 1 h (physical hydrogels) or 2 h (chemical hydrogels in the presence of glutaraldehyde). (c) Schematic representation of the diffusion of dextrans of different MW within ELP hydrogels with mesh size ξ. (d) Snapshots of a representative photobleaching experiment for ELP hydrogels (15 wt %) at 37 °C using FITC-dextrans with MWs of 40 and 150 kDa. (e) Fluorescence recovery curves (*n=5*) for hydrogel samples at 37 °C for 40 kDa dextrans (left, hydrodynamic radius *r* ∼ 4.5 nm) and 150 kDa dextrans (right, *r* ∼ 8.5 nm). (f) Ratio between the diffusivity *D* of 40 and 150 kDa dextrans in ELP hydrogels (15 wt %, 37 °C) and the predicted diffusivity *D*_0_ from eq 6 in Milli-Q water (1 mg/mL).

FTIR spectra were used to assess the secondary structure of flash-frozen and freeze-dried ELPs in solution (4 °C) and hydrogel (37 °C) states (15 wt %). To do so, we inspected the amide I region of the spectra (1600–1700 cm^–1^). This region is commonly used for quantitative determination of the secondary structure in protein materials.^80^ We found a modest increase in ordered structures after gelation (from ∼26–32% to ∼32–40%) (Figure 3a,b), as shown by the increase in the peak associated with β-sheets at 1620 cm^–1^. This increase was less pronounced in chemical hydrogels (Table S5), likely because covalent cross-links hindered the formation of ordered structures in the ELP network at 37 °C. The modest increase in order observed in physical hydrogels likely originated from IPAVG blocks, which can lead to more structural order above *T*_t_.^81^ Despite the modest shift toward more order after gelation, the hydrogels remained mostly disordered (Figure S16). The high elastic modulus of E_AE,triblock_, E_AI,triblock_, or chemical hydrogels, despite their lack of order, indicates that nanoscale order is not a requirement for mechanical reinforcement in ELP materials.

To assess the microstructure of the hydrogels, we first estimated their mesh size from their measured elastic modulus *G*′ using rubber elasticity theory^72^ (eq 4). The predicted mesh sizes ranged from 4.5 ± 0.7 nm (E_KE_) to 15.0 ± 4.2 nm (E_AI_) (Table 2). We also independently probed the mesh size experimentally by performing FRAP diffusion measurements for fluorescein isothiocyanate labeled dextran tracers of different sizes (MW of 40 and 150 kDa, radii of ca. 4.5 and 8.5 nm, respectively) within ELP hydrogels incubated at 37 °C (Figure 3c,d) for 1 h (physical hydrogels) or 2 h (chemical hydrogels). Glutaraldehyde used to prepare chemical hydrogels did not interfere with the diffusion of dextrans (Figure S17). We found that the diffusion of 40 kDa dextrans was unhindered (i.e., identical to that of a pure dextran solution in Milli-Q water) for E_AI_. For the rest of our ELP library, diffusion of 40 kDa dextrans was hindered by the presence of the hydrogel network. The larger 150 kDa dextrans showed an even slower fluorescence recovery (Figure 3e), in this case also within E_AI_ networks. Fitting the fluorescence recovery data to a single exponential (Figure S18) allowed us to estimate the diffusivity of dextrans in the different ELP networks (Table 2). This value was normalized by the diffusivity *D*_0_ in solution calculated using the Stokes–Einstein relation (73.0 and 38.6 μm/s^2^ for 40 and 150 kDa dextrans, respectively; Figure 3f). Generally, chemical hydrogels led to lower diffusivities. Given that the diffusion of 150 kDa dextrans (hydrodynamic radius of 8.5 nm) was hindered in all samples, this suggests that the mesh sizes were below 17 nm. This estimate is consistent with the mesh sizes calculated from the gel’s elastic modulus via rubber elasticity theory. The diffusion of dextrans was similar in all samples, despite the variations in the amino acid sequence. A similar phenomenon was reported in networks formed by ELPs fused to helical partially ordered polypeptides,^44^ where MW, helical percentage, or helix sequence did not impact their void volume.

**Table 2:** Microstructural Characterization of ELP Hydrogels: Mesh Size as Calculated from the Gel Elastic Modulus *G*′ Following Eq 4; Fluorescence Recovery Rate *k*_FRAP_ Obtained by Fitting FRAP Data and Diffusion Constant *D*

		dextrans 40 kDa		dextrans 150 kDa	
ELP design	ξ (nm)	*k*_FRAP_(s^–1^)	*D* (μm^2^/s)	*k*_FRAP_(s^–1^)	*D* (μm^2^/s)
solution 1mg/mL		0.179 ± 0.002	73.0 ± 0.8	0.138 ± 0.001	38.6 ± 0.3
E_AE,triblock_	9.9 ± 0.8	0.115 ± 0.001	47.4 ± 0.4	0.094 ± 0.001	26.7 ± 0.3
E_KE_ + GA	4.5 ± 0.7	0.051 ± 0.001	21.0 ± 0.4	0.057 ± 0.000	16.2 ± 0.3
E_AI_	15.0 ± 4.2	0.186 ± 0.002	76.7 ± 0.4	0.129 ± 0.002	36.7 ± 0.6
E_AI,triblock_	6.0 ± 0.3	0.087 ± 0.001	35.9 ± 0.4	0.038 ± 0.008	10.8 ± 2.3
E_KI_ + GA	4.8 ± 0.6	0.063 ± 0.001	26.0 ± 0.4	0.036 ± 0.002	10.2 ± 0.6

We note that the mesh size estimates for hydrated hydrogels obtained using eq 4 or from probe diffusivity measurements with FRAP differed by 3 orders of magnitude from the average pore sizes measured via SEM on dried hydrogels (5.2 ± 1.1 μm for E_AE,triblock_, 2.7 ± 0.7 μm for E_KE_+GA, 1.5 ± 0.4 μm for E_AI,triblock_, or 0.8 ± 0.3 μm for E_KI_ + GA; determination of the average pore size was not possible for E_AI_ due to its highly heterogeneous structure; Figure S19). We hypothesize that the large pores observed via SEM are artifacts of the drying process needed to image the samples with this technique. Thus, our results show that techniques that probe hydrogels in hydrated, nonperturbed conditions are required to obtain reliable microstructural data for ELP hydrogels.

## Conclusion

Analysis of the multidimensional data set of molecular properties obtained via short MD simulations allowed us to develop a predictive model for the phase behavior of ELPs. This model unambiguously classified a library of 8 ELPs by their presence or absence of LCST phase behavior below 37 °C. It did so without the need for computationally expensive long simulation times or advanced sampling methods. Experimental characterization indicated that ELPs with physical cross-linking of IPAVG blocks in triblock arrangements formed stiff and reversible hydrogels at physiological temperature. Nonpolar guest amino acids (isoleucine) facilitated the formation of stiffer hydrogel networks, albeit with a higher viscous response. Chemical hydrogels attained the highest elastic response, but without thermoresponsiveness. Triblock sequences exhibited hysteresis upon cooling that was not observed for multiblock arrangements. Silk-like GAGAGS blocks hindered the formation of hydrogel networks, highlighting the need for larger ELP regions (more than the five VPGXG repeats used here) between silk-blocks to enable self-assembly and network formation. Theory and experiments also demonstrated the need to probe ELP hydrogels in a hydrated and unperturbed manner to obtain reliable information about the network structure. The hydrogels displayed mesh sizes below 17 nm at ELP concentrations of 15 wt %, with little dependence on the ELP sequence.

Overall, our results reveal multiple handles to orthogonally control the features of ELP hydrogels via block selection and sequence organization: chain hydrophilicity/hydrophobicity and/or block distribution can be used to control the viscoelastic properties and *T*_t_, whereas polypeptide concentration defines the network permeability. Furthermore, we showed the potential of MD simulations to mitigate the costs of developing *de novo* ELP designs by predicting their LCST phase behavior before synthesizing them. This opens the possibility to explore a larger sequence space at a faster pace, where it will be interesting to test the robustness of the model for different molecular weights and new building blocks.

## Supporting Information Available

Sequences for MD simulations, molecular properties and additional data analysis of the simulation results, FTIR data, agarose and SDS PAGE gels, additional rheological characterization, individual FRAP curves, and SEM images of the hydrogels (PDF)
