# Correction to “Sequence Control of the Self-Assembly of Elastin-Like Polypeptides into Hydrogels with Bespoke Viscoelastic and Structural Properties”

**DOI:** 10.1021/acs.biomac.5c01225

**Published:** 2025-08-11

**Authors:** Diego López Barreiro, Abel Folch-Fortuny, Iain Muntz, Jens C. Thies, Cees M. J. Sagt, Gijsje H. Koenderink

We would like to report a typographical error in our previously publication. The error concerns the distribution of building blocks for the polypeptides E_KE_ and E_KI_ in Table 1.

The sequences were incorrectly reported as

E_KE_: (VPGKG)_12_[(VPGVG)_2_(VPGEG)­(VPGVG)_2_]_12_(VPGKG)_12_

E_KI_: (VPGKG)_12_[(VPGVG)_2_(VPGIG)­(VPGVG)_2_]_12_(VPGKG)_12_

The correct sequences are

E_KE_: [(VPGKG)­(VPGVG)_2_(VPGEG)­(VPGVG)_2_(VPGKG)]_12_

E_KI_: [(VPGKG)­(VPGVG)_2_(VPGIG)­(VPGVG)_2_(VPGKG)]_12_

This error in the building block distribution was also propagated to Table S1, which contains shorter variants of these sequences used for molecular dynamics (MD) simulations. The correct sequences in Table S1 are

E_KE_: [(VPGKG)­(VPGVG)_2_(VPGEG)­(VPGVG)_2_(VPGKG)]_5_

E_KI_: [(VPGKG)­(VPGVG)_2_(VPGIG)­(VPGVG)_2_(VPGKG)]_5_

This error is limited to the sequences of E_KE_ and E_KI_ in Table 1 and Table S1. The hydropathy plots shown for those two polypeptides in Figure 1a are correct. Furthermore, we have verified that the sequences used in the MD simulations for E_KE_ and E_KI_ correspond to the corrected sequences provided above for Table S1.

These typographical errors do not affect the results or conclusions of the paper. We apologize for any confusion that this may have caused.

