# Supplementary material for: Sequence Control of the Self-Assembly of Elastin-Like Polypeptides into Hydrogels with Bespoke Viscoelastic and Structural Properties
Source: Biomacromolecules. 2022 Dec 14;24(1):489–501. doi: 10.1021/acs.biomac.2c01405 (PMC9832484; doi:10.1021/acs.biomac.2c01405)
Supplement: Supplementary file 1 — bm2c01405_si_001.pdf [file bm2c01405_si_001.pdf]

SUPPORTING INFORMATION

Sequence control of the self-assembly of elastin-like polypeptides into hydrogels with bespoke viscoelastic and structural properties

Diego López Barreiro[1], Abel Folch-Fortuny[2], Iain Muntz[3],

Jens C. Thies[4*], Cees M. J. Sagt[1*], Gijsje H. Koenderink[3*]

[1]*DSM Biosciences and Process Innovation, DSM, Alexander Fleminglaan 1, 2613 AX Delft, The Netherlands*

[2]*DSM Biodata and Translation, DSM, Alexander Fleminglaan 1, 2613 AX Delft, The Netherlands*

[3]*Department of Bionanoscience, Kavli Institute of Nanoscience Delft,*

Delft University of Technology, Van der Maasweg 9, 2629 HZ Delft,

The Netherlands

[4]*DSM Biomedical, DSM, Urmonderbaan 22, 6160 BB, Geleen, The Netherlands*

**Corresponding authors: Jens.Thies@dsm.com, Cees.Sagt@dsm.com, G.H.Koenderink@tudelft.nl*

Table S1 *– ELP sequences used for MD simulations.*

Design	Sequence
E_{AE}	$[(IPAVG)(VPGVG)_2(VPGEG)(VPGVG)_2(IPAVG)]_5$
$E_{AE,triblock}$	$(IPAVG)_5[(VPGVG)_2(VPGEG)(VPGVG)_2]_5(IPAVG)_5$
SE_E	$[(GAGAGS)(VPGVG)_2(VPGEG)(VPGVG)_2(GAGAGS)]_5$
E_{KE}	$(VPGKG)_5[(VPGVG)_2(VPGEG)(VPGVG)_2]_5(VPGKG)_5$
E_{AI}	$[(IPAVG)(VPGVG)_2(VPGIG)(VPGVG)_2(IPAVG)]_5$
$E_{AI,triblock}$	$(IPAVG)_5[(VPGVG)_2(VPGIG)(VPGVG)_2]_5(IPAVG)_5$
SE_I	$[(GAGAGS)(VPGVG)_2(VPGIG)(VPGVG)_2(GAGAGS)]_5$
E_{KI}	$(VPGKG)_5[(VPGVG)_2(VPGIG)(VPGVG)_2]_5(VPGKG)_5$

Table S2 *– Full list of molecular properties extracted from the last 50 ns of each MD simulation replica.*

Molecular property	***Units***	***Description***
H-bonds	*[-]*	*Count of the number of intrapeptide hydrogen bonds.*
H-bonds per residue	*[-]*	*Count of the number of intrapeptide hydrogen bonds, normalized by the number of residues in the ELP (185 for SE_E and SE_I, 175 for the rest).*
H-bonds crosslinking blocks	*[-]*	*Count of the number of intrapeptide hydrogen bonds formed by amino acids in the crosslinking blocks IPAVG, GAGAGS or VPGKG in the ELP.*
H-bonds crosslinking blocks per residue	*[-]*	*Count of the number of intrapeptide hydrogen bonds formed by amino acids in the crosslinking blocks IPAVG, GAGAGS or VPGKG in the ELP, normalized by the number of residues in the crosslinking blocks (60 for SE_E and SE_I, 50 for the rest).*
H-bonds water	*[-]*	*Count of the number of hydrogen bonds between the ELP and the solvent (water).*
H-bonds water per residue	*[-]*	*Count of the number of hydrogen bonds between the ELP and the solvent (water), normalized by the number of residues in the ELP (185 for SE_E and SE_I, 175 for the rest).*
H-bonds water crosslinking blocks	*[-]*	*Count of the number of hydrogen bonds between crosslinking blocks IPAVG, GAGAGS or VPGKG in the ELP and the solvent (water).*
H-bonds water crosslinking blocks per residue	*[-]*	*Count of the number of hydrogen bonds between crosslinking blocks IPAVG, GAGAGS or VPGKG in the ELP and the solvent (water), normalized by the number of residues in the crosslinking blocks (60 for SE_E and SE_I, 50 for the rest).*
SASA hydrophobic	*[$Å^2$]*	*Solvent accessible surface area for hydrophobic amino acids.*
SASA hydrophobic per residue	*[$Å^2$]*	*Solvent accessible surface area for hydrophobic amino acids, normalized by the number of residues in the ELP (185 for SE_E and SE_I, 175 for the rest).*

SASA hydrophilic	*[Å^2]*	*Solvent accessible surface area for hydrophilic amino acids.*
SASA hydrophilic per residue	*[Å^2]*	*Solvent accessible surface area for hydrophilic amino acids, normalized by the number of residues in the ELP (185 for SE_E and SE_I, 175 for the rest).*
SASA total per residue	*[Å^2]*	*Total solvent accessible surface area (hydrophobic+hydrophilic), normalized by the number of residues in the ELP (185 for SE_E and SE_I, 175 for the rest).*
SASA total crosslinking blocks	*[Å^2]*	*Total solvent accessible surface area (hydrophobic+hydrophilic) for the crosslinking blocks IPAVG, GAGAGS or VPGKG in the ELP.*
SASA total crosslinking blocks per residue	*[Å^2]*	*Total solvent accessible surface area (hydrophobic+hydrophilic) for the crosslinking blocks IPAVG, GAGAGS or VPGKG in the ELP, normalized by the number of residues in the crosslinking blocks (60 for SE_E and SE_I, 50 for the rest).*
r_{gyr}	*[Å]*	*Radius of gyration of the ELP, tracks its shape change throughout the simulation.*
β-sheet %	*[%]*	*% of the ELP sequence in β-sheet conformation, as calculated by the STRIDE algorithm.*
Turn %	*[%]*	*% of the ELP sequence in turn conformation, as calculated by the STRIDE algorithm.*
Helix %	*[%]*	*% of the ELP sequence in helix conformation, as calculated by the STRIDE algorithm.*
Random coil %	*[%]*	*% of the ELP sequence in random coil conformation, as calculated by the STRIDE algorithm.*
RMSD	*[Å]*	*Root mean squared deviation of atomic positions, tracks the conformational changes of the ELP throughout the simulation.*
Hydration waters	*[-]*	*Counts the number of hydration water molecules surrounding the ELP within 3.15 Å.*
Hydration waters per residue	*[-]*	*Counts the number of hydration water molecules surrounding the ELP within 3.15 Å, normalized by the number of residues in the ELP (185 for SE_E and SE_I, 175 for the rest).*
Hydration waters crosslinking blocks	*[-]*	*Counts the number of hydration water molecules within 3.15 Å from crosslinking blocks IPAVG, GAGAGS or VPGKG in the ELP.*
Hydration waters crosslinking blocks per residue	*[-]*	*Counts the number of hydration water molecules within 3.15 Å from crosslinking blocks IPAVG, GAGAGS or VPGKG in the ELP, normalized by the number of residues in the crosslinking blocks (60 for SE_E and SE_I, 50 for the rest).*
Electrostatic energy	*[kcal/mol]*	*Electrostatic energy (in kJ/mol) between the ELP and the solvent (water), as calculated using the NAMD Energy plugin.*
van der Waals energy	*[kcal/mol]*	*Van der Waals energy (in kJ/mol) between the ELP and the solvent (water), as calculated using the NAMD Energy plugin.*

***Table S3** – Total amino acid composition (in mol %) of ELPs as obtained by the Waters Accq Tag method (“Experimental”). These values are compared to the theoretical (“Theoretical”) composition of each ELP.*

Design		His	Ser	Arg	Gly	Asx	Glx	Thr	Ala	Pro	Lys	Met	Val	Ile	Leu	Phe
E_{AE}	Experimental	-	0.1 ± 0.1%	0.1 ± 0.0%	34.0 ± 0.0%	0,1 ± 0,0%	3.0 ± 0.0%	0.1 ± 0.0%	5.5 ± 0.0%	19.9 ± 0.1%	-	0.1 ± 0.0%	31.5 ± 0.0%	5.6 ± 0.0%	0.1 ± 0.0%	-
	Theoretical	-	-	-	34.2%	-	2.9%	-	5.7%	20.0%	-	0.2%	31.4%	5.7%	-	-
$E_{AE,triblock}$	Experimental	-	0.1 ± 0.0%	0.1 ± 0.0%	33.6 ± 0.1%	0,3 ± 0,0%	3.1 ± 0.0	0.1 ± 0.0%	5.5 ± 0.0%	19.7 ± 0.0%	0,1 ± 0,0%	0.2 ± 0.0%	31.3 ± 0.0%	5.5 ± 0.0%	0.2 ± 0.0%	0.1 ± 0.0%
	Theoretical	-	-	-	34.2%	-	2.9%	-	5.7%	20.0%	-	0.2%	31.4%	5.7%	-	-
SE_{AE}	Experimental	-	5.0 ± 0.0%	0.2 ± 0.1%	43.6 ± 0.3%	0.4 ± 0,1%	3.0 ± 0.1%	0.2 ± 0.0%	10.6 ± 0.0%	13.4 ± 0.1%	0.3 ± 0.1%	0.2 ± 0.0%	23.8 ± 0.1%	0.2 ± 0.0%	0.2 ± 0.1%	0.1 ± 0.0%
	Theoretical	-	5.4%	-	43.1%	-	2.7%	-	10.8%	13.5%	-	0.2%	24.3%	-	-	-
E_{KE}	Experimental	-	0.1 ± 0.1%	-	39.6 ± 0.0%	0,1 ± 0,0%	3.0 ± 0.0%	-	0.1 ± 0.0%	19.9 ± 0.0%	5.7 ± 0.0%	-	31.4 ± 0.1%	-	-	-
	Theoretical	-	-	-	39.9%	-	2.9%	-	-	20.0%	5.7%	0.2%	31.4%	-	-	-
E_{AI}	Experimental	0.1 ± 0.1%	0.1 ± 0.1%	-	34.0 ± 0.2%	0.1 ± 0.0%	-	-	5.5 ± 0.0%	20.2 ± 0.1%	-	0.1 ± 0.0%	31.6 ± 0.1%	8.4 ± 0.0	-	-
	Theoretical	-	-	-	34.2%	-	-	-	5.7%	20.0%	-	0.2%	31.4%	8.6%	-	-
$E_{AI,triblock}$	Experimental	-	-	-	34.3 ± 0.0%	-	-	-	5.4 ± 0.0%	20.0 ± 0.1%	-	0.1 ± 0.0%	31.8 ± 0.0%	8.3 ± 0.1%	-	-
	Theoretical	-	-	-	34.2%	-	-	-	5.7%	20.0%	-	0.2%	31.4%	8.6%	-	-
SE_{AI}	Experimental	-	4.8 ± 0.0%	-	43.4 ± 0.1%	-	0.1 ± 0.0%	-	10.5 ± 0.0%	13.6 ± 0.0%	-	-	24.7 ± 0.0%	2.7 ± 0.0%	-	-
	Theoretical	-	5.4%	-	43.1%	-	-	-	10.8%	13.5%	-	0.2%	24.3%	2.7%	-	-
E_{KI}	Experimental	-	0.1 ± 0.1%	-	39.8 ± 0.0%	-	-	-	-	20.1 ± 0.1%	5.6 ± 0.1%	-	31.5 ± 0.1%	2.8 ± 0.0%	-	-
	Theoretical	-	-	-	39.9%	-	-	-	-	20.0%	5.7%	0.2%	31.4%	2.9%	-	-

Table S4 *–Regression coefficients (average ± st error) obtained via double cross validation for the PLS-DA model. High values in MD simulations for molecular properties with large positive regression coefficients increase the likelihood of LCST behavior. Conversely, high values for molecular properties with large negative regression coefficients decrease the likelihood of LCST behavior.*

**Molecular property**	**Regression coefficient**
SASA hydrophobic (per residue)	0.049 ± 0.004
SASA hydrophobic	0.048 ± 0.004
β-sheet %	0.039 ± 0.002
Electrostatic energy	0.028 ± 0.003
Random coil %	0.005 ± 0.003
SASA total (per residue)	-0.002 ± 0.003
H-bonds intraprotein crosslinking blocks (per residue)	-0.008 ± 0.003
H-bonds water (per residue)	-0.009 ± 0.005
Hydration waters (per residue)	-0.009 ± 0.003
van der Waals energy	-0.012 ± 0.003
H-bonds (per residue)	-0.014 ± 0.002
SASA total crosslinking blocks (per residue)	-0.015 ± 0.004
r_{gyr}	-0.016 ± 0.002
H-bonds water	-0.016 ± 0.004
H-bonds intraprotein crosslinking blocks	-0.017 ± 0.003
H-bonds	-0.018 ± 0.002
Hydration waters	-0.020 ± 0.003
Helix %	-0.020 ± 0.004
Turn %	-0.021 ± 0.003
SASA total crosslinking blocks	-0.030 ± 0.002
RMSD	-0.030 ± 0.002
Hydration waters crosslinking blocks (per residue)	-0.032 ± 0.001
Hydration waters crosslinking blocks	-0.038 ± 0.001
Hbonds water crosslinking blocks (per residue)	-0.041 ± 0.001
Hbonds water crosslinking blocks	-0.047 ± 0.001
SASA hydrophilic (per residue)	-0.051 ± 0.002
SASA hydrophilic	-0.053 ± 0.002

Table S5 *– Secondary structure of freeze-dried and cryo-fractured ELPs from a 15 wt % solution in milliQ water below T_t (top) and as a hydrogel above T_t (bottom), as obtained by deconvoluting the amide I peak (n=2).*

Solution state	$E_{AE,triblock}$	E_{KE}	E_{AI}	$E_{AI,triblock}$	E_{KI}
**β-sheet [%]**	31.8 ± 0.9	28.2 ± 1.7	30.6 ± 0.0	27.2 ± 1.9	26.0 ± 0.1
**Random Coil/Helix [%]**	64.0 ± 1.0	66.1 ± 1.5	64.4 ± 0.4	67.0 ± 1.8	67.8 ± 0.3
**Turn [%]**	4.2 ± 0.0	5.7 ± 0.2	5.0 ± 0.4	6.2 ± 0.1	6.2 ± 0.1

Hydrogel state	$E_{AE,triblock}$	E_{KE}	E_{AI}	$E_{AI,triblock}$	E_{KI}
**β-sheet [%]**	40.1 ± 0.3	31.8 ± 1.6	37.4 ± 3.8	34.4 ± 1.6	31.7 ± 0.8
**Random Coil/Helix [%]**	57.3 ± 0.2	61.4 ± 0.0	59.3 ± 3.7	61.5 ± 1.5	63.9 ± 0.5
**Turn [%]**	2.6 ± 0.1	4.2 ± 0.2	3.4 ± 0.0	4.2 ± 0.1	4.5 ± 0.3

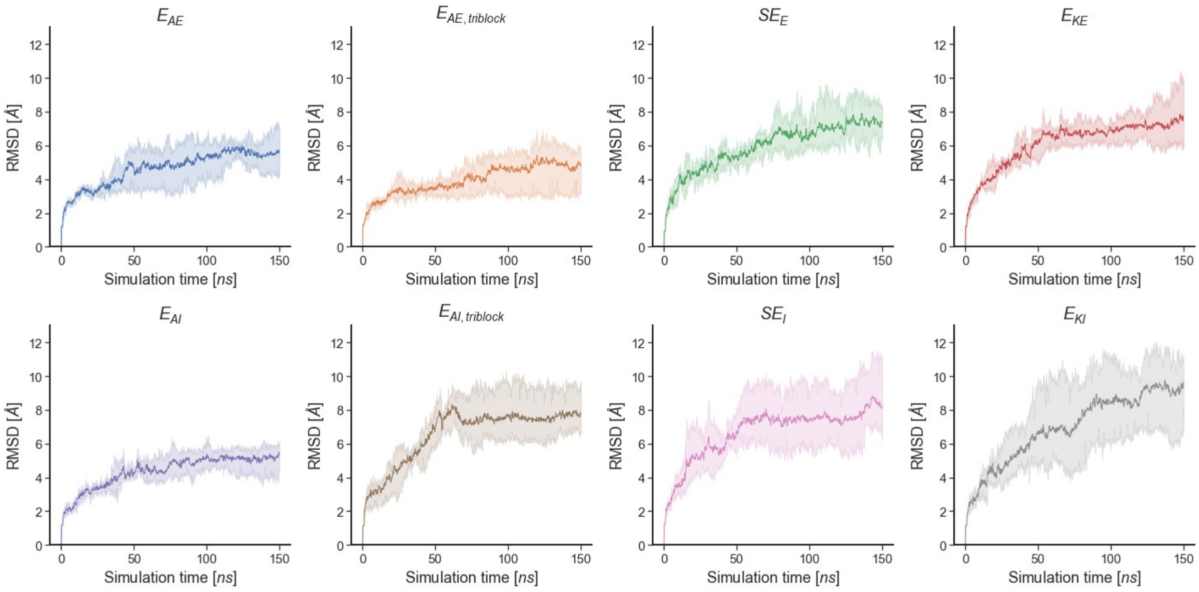

Figure S1 *– Time evolution of the root mean squared displacement for the different ELPs considered in this work during MD simulations (n=3). In each case, a plateau is reached within the simulation time. Shaded area represents standard deviation over 3 runs.*

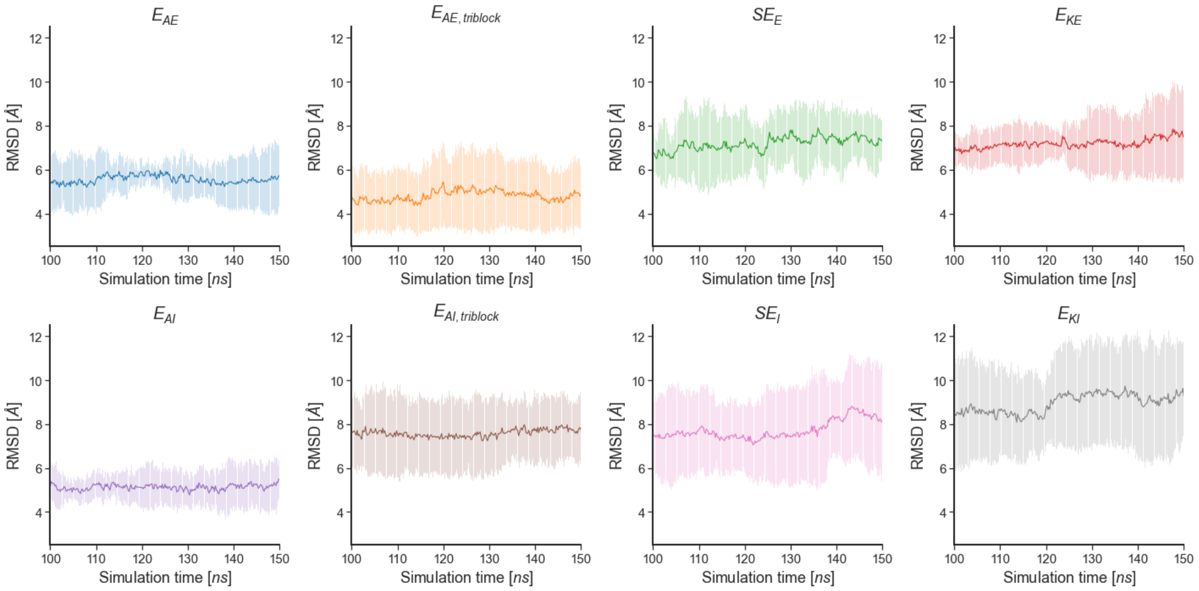

Figure S2*– Time evolution of the root mean squared displacement of atomic positions of the different ELPs considered in this work over the last 50 ns during MD simulations (n=3). Shaded area represents standard deviation over 3 runs.*

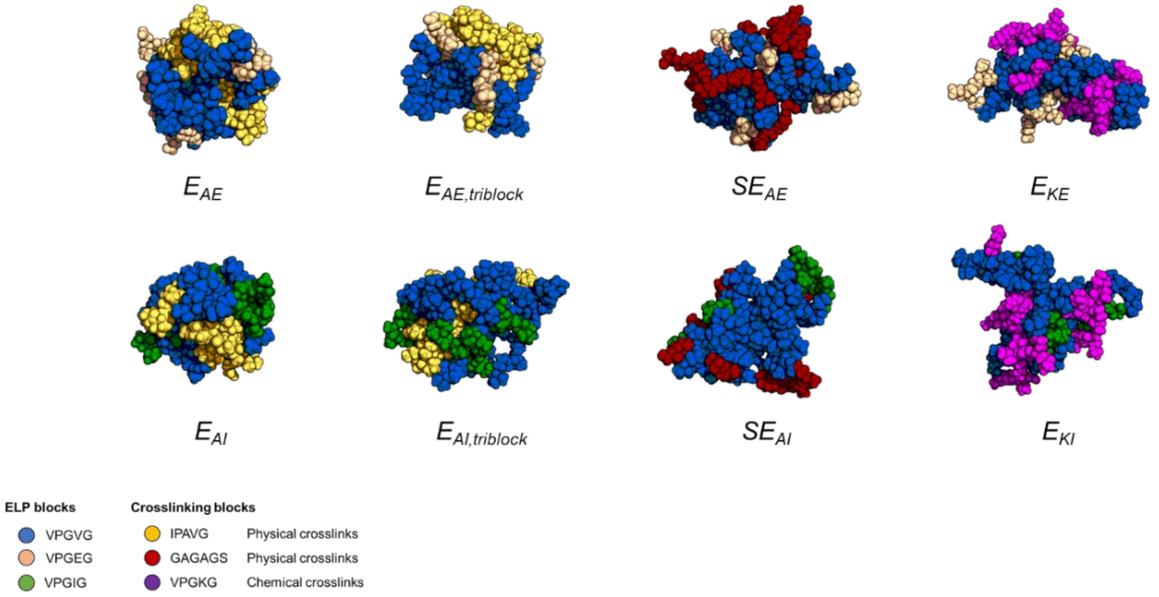

Figure S3 *– Representative snapshots of the final structures of each ELP, color-coded to indicate the location of the different building blocks (see legend). The color code used is the same as in Figure 1a in the main text.*

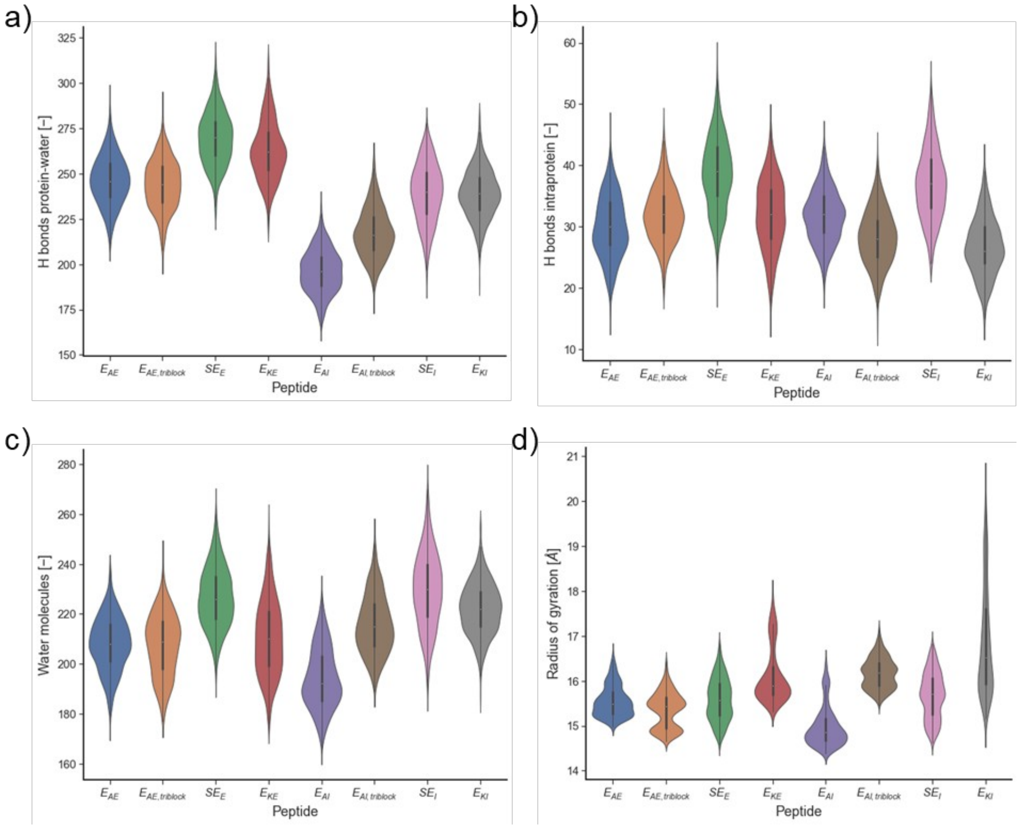

Figure S4 *– Distribution of (a) ELP-water hydrogen bonds, (b) intra-ELP hydrogen bonds, (c) water molecules in the hydration layer, and (d) radius of gyration of ELPs during the last 50 ns of MD simulations (n=3).*

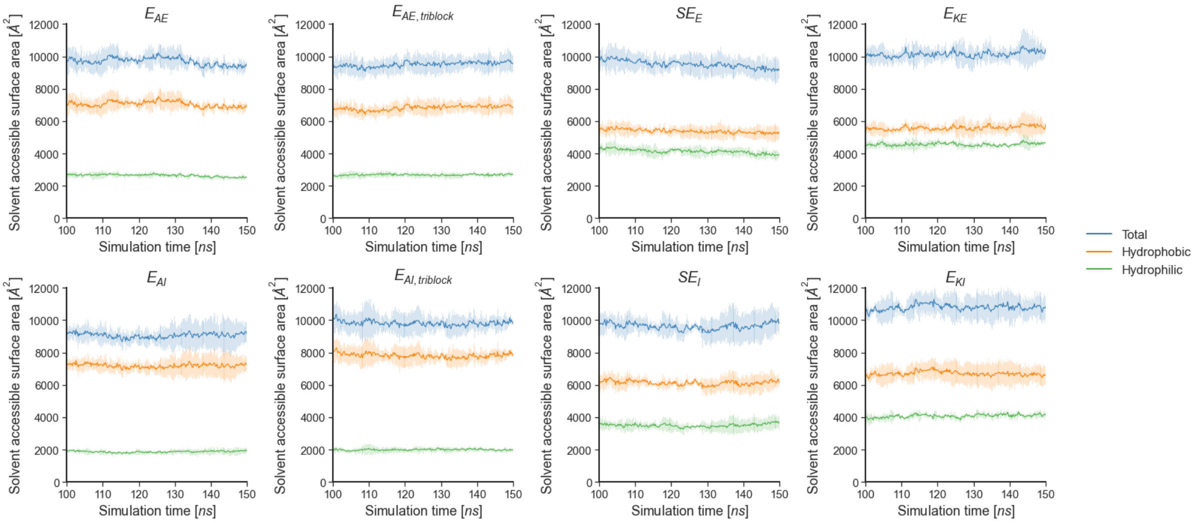

Figure S5 *- Time evolution of the solvent accessible surface area (SASA) for the different ELPs considered in this work over the last 50 ns during MD simulations (n=3). The data shows the evolution of the total SASA (blue), as well as the SASA of hydrophobic (orange) and hydrophilic (green) parts of the ELPs. Shaded area represents standard deviation over 3 runs.*

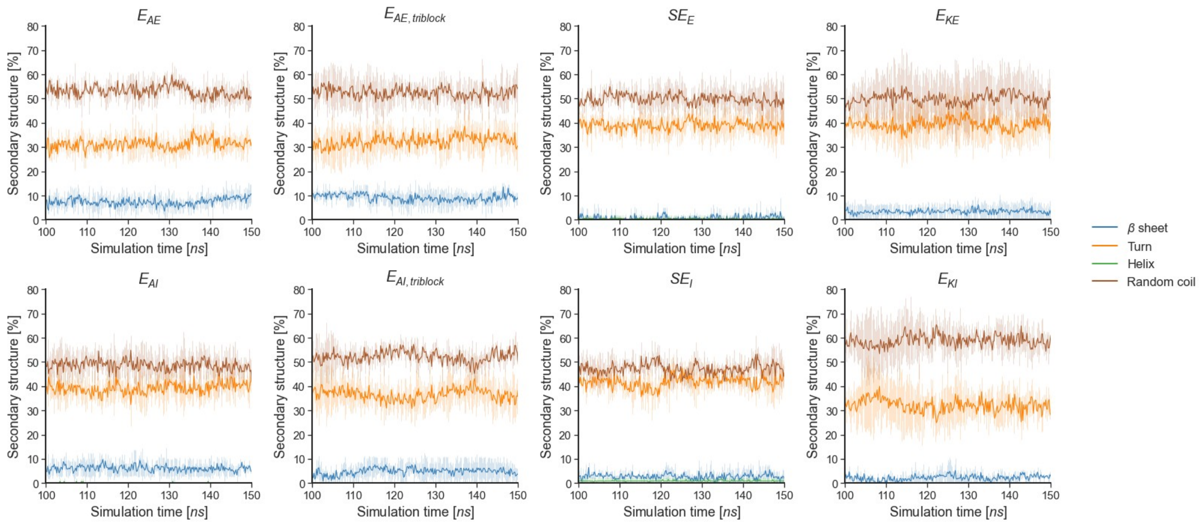

Figure S6 *- Time evolution of the secondary structure for the different ELPs considered in this work over the last 50 ns during MD simulations (n=3). Shaded area represents standard deviation over 3 runs.*

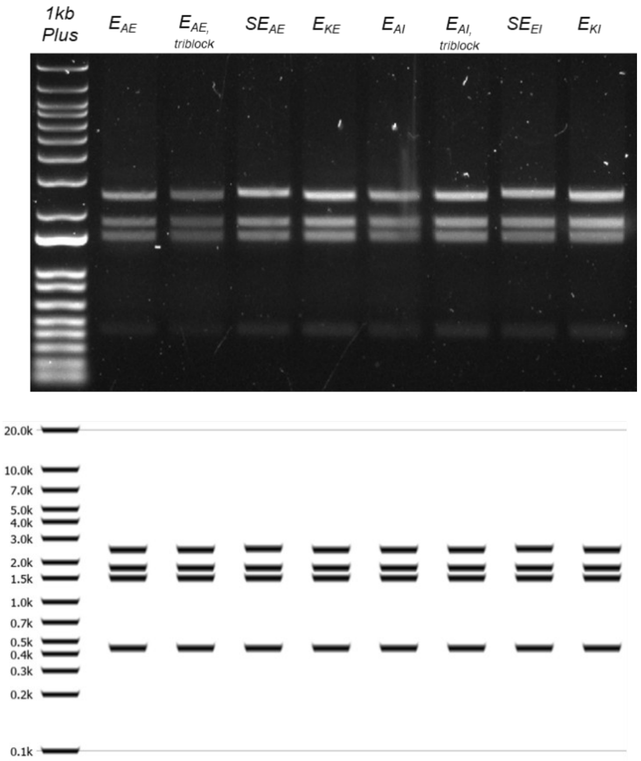

Figure S7 *– Plasmid digestion in 0.5% agarose gel, depicting the correct insertion of the vectors containing the different ELP designs. The left lane shows the DNA standard 1kb+ ladder (New England Biolabs).*

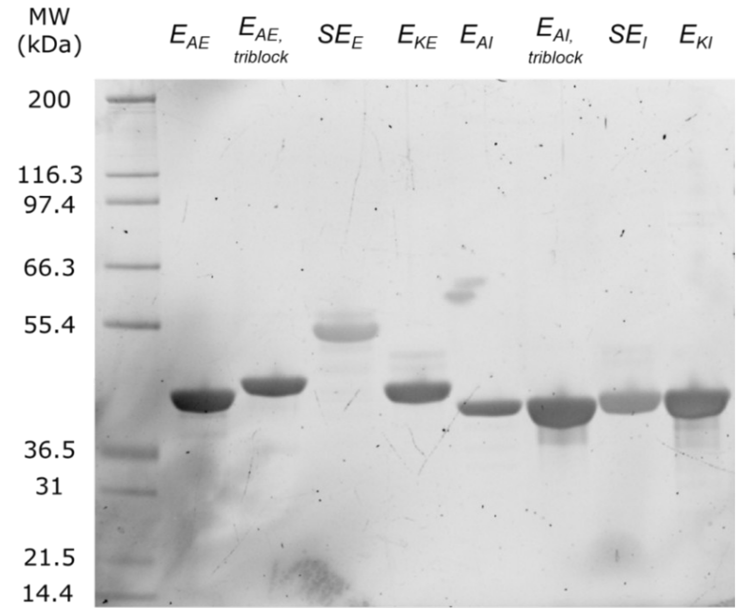

Figure S8 *– SYPRO® Red-stained SDS-PAGE gel of the purified ELPs. The left lane is the Mark12 unstained protein MW standard (Thermo Fisher Scientific). Lanes 2 to 9 show the different ELPs. It is a well-documented phenomenon that the theoretical MW of ELPs and their position in the SDS-PAGE gel can show deviations of up to 20%, especially when containing alanine residues [1]. The purity of the samples was >98%, as determined by Total Amino Acid analysis (Table S1).*

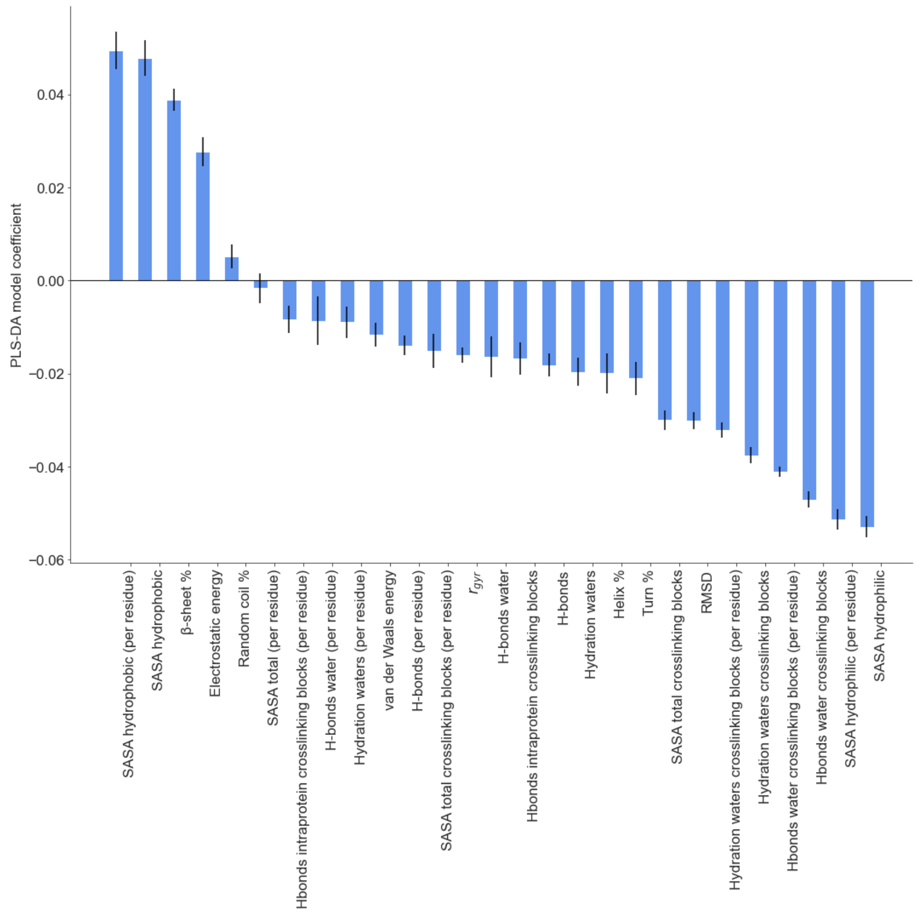

Figure S9 *- Average regression coefficients for the PLS-DA model, including error bars with the standard errors produced via double cross-validation. Each regression coefficient corresponds to molecular properties sampled from MD simulations.*

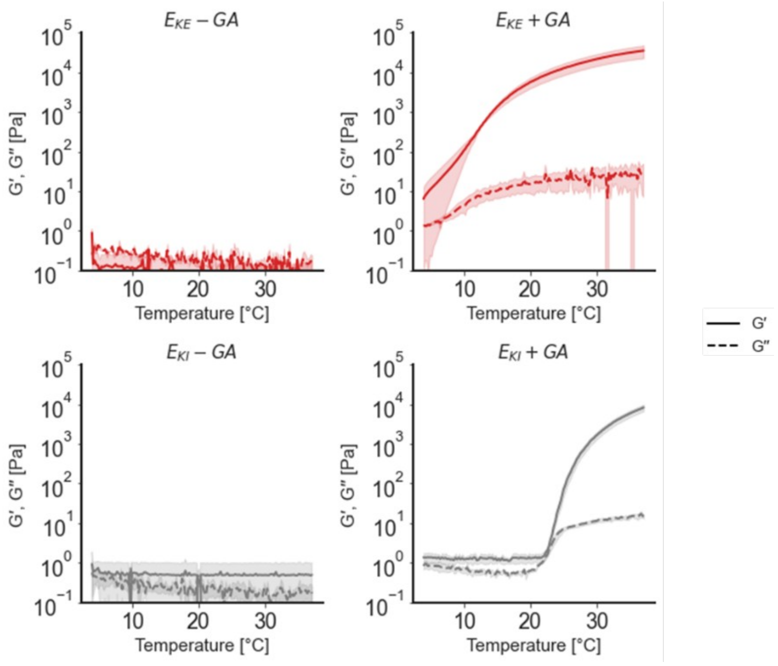

Figure S10 *– Rheological properties of lysine-containing ELPs in milliQ water (E_{KE} and E_{KI}) without (left column) and with (right column) glutaraldehyde (GA), showing the elastic and viscous modulus as a function of temperature (f=1 Hz, γ=0.3%) (n=2).*

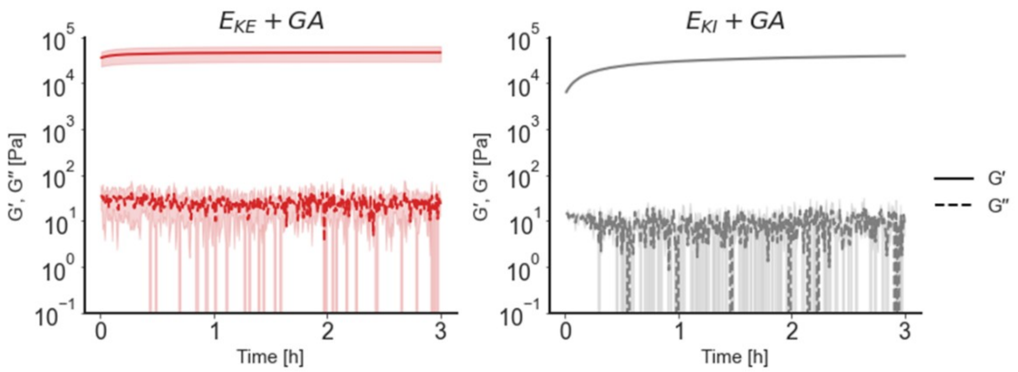

Figure S11 *- Time sweeps (3 h, 37 °C) of solutions of lysine-containing ELPs in milliQ water (E_{KE} and E_{KI}) after the addition of glutaraldehyde and incubation on ice for 10 min, showing the elastic and viscous shear moduli (f=1 Hz, γ=0.3%) (n=2).*

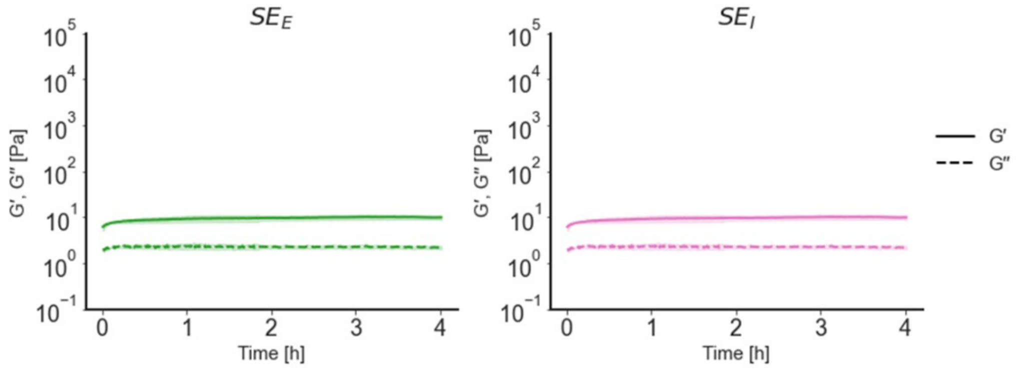

Figure S12 *– Time sweeps (4 h, 37 °C) of solutions in milliQ water of ELPs containing silk-like blocks (SE_E and SE_I) at 15 wt %, γ=0.3%, f=1 Hz (n=2).*

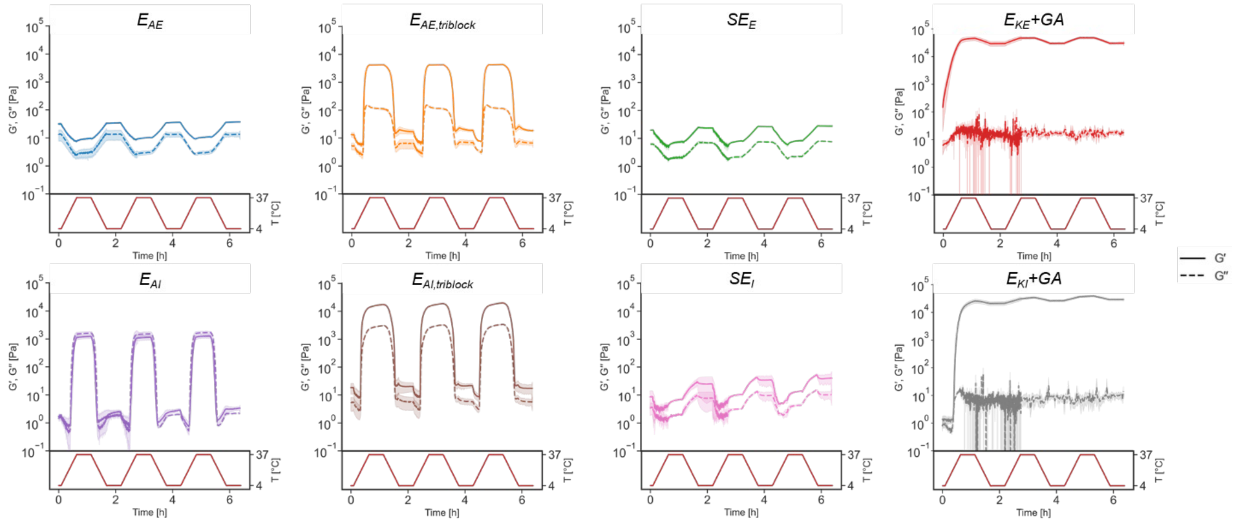

Figure S13 *- Thermal cycling (3 heating-cooling cycles with 30 min of resting time between temperature ramps) of ELP solutions (15 wt % in milliQ water, with a 40:1 ELP:glutaraldehyde ratio for E_{KE} and E_{KI}) at a rate of 1 °C/min. Measurements were taken at f=1 Hz, γ=0.3% (n=2).*

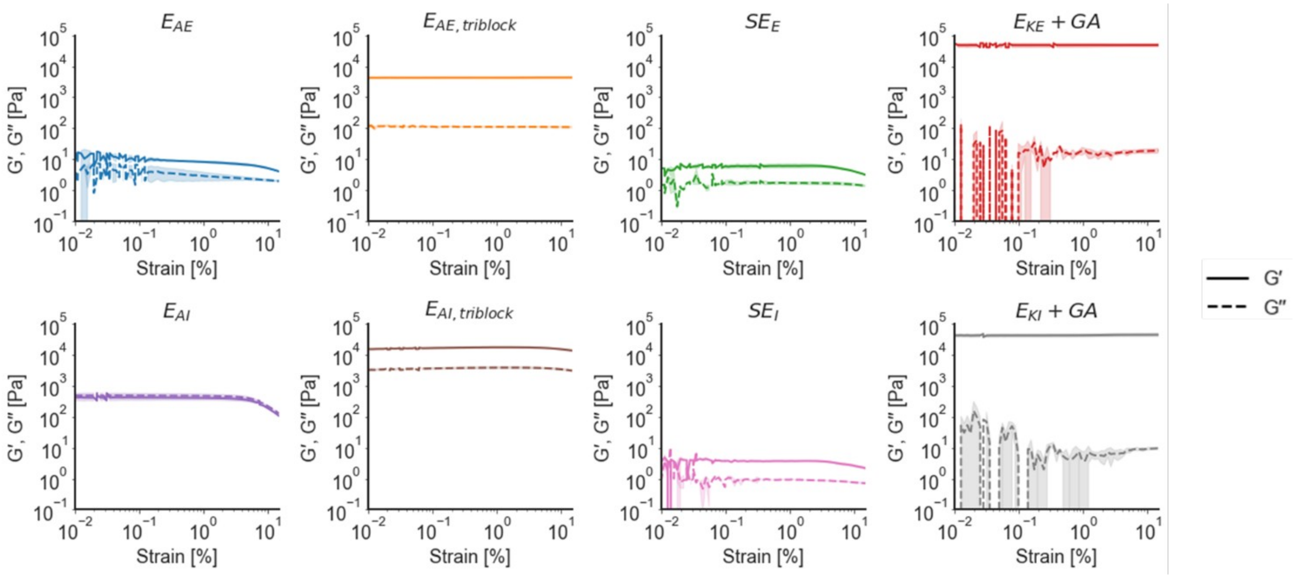

***Figure S14** – Amplitude sweeps (γ=0.01-15 %, f=1 Hz) of ELP solutions (15 wt % in milliQ water, with a 40:1 ELP:glutaraldehyde ratio for E_{KE} and E_{KI}) (n=2).*

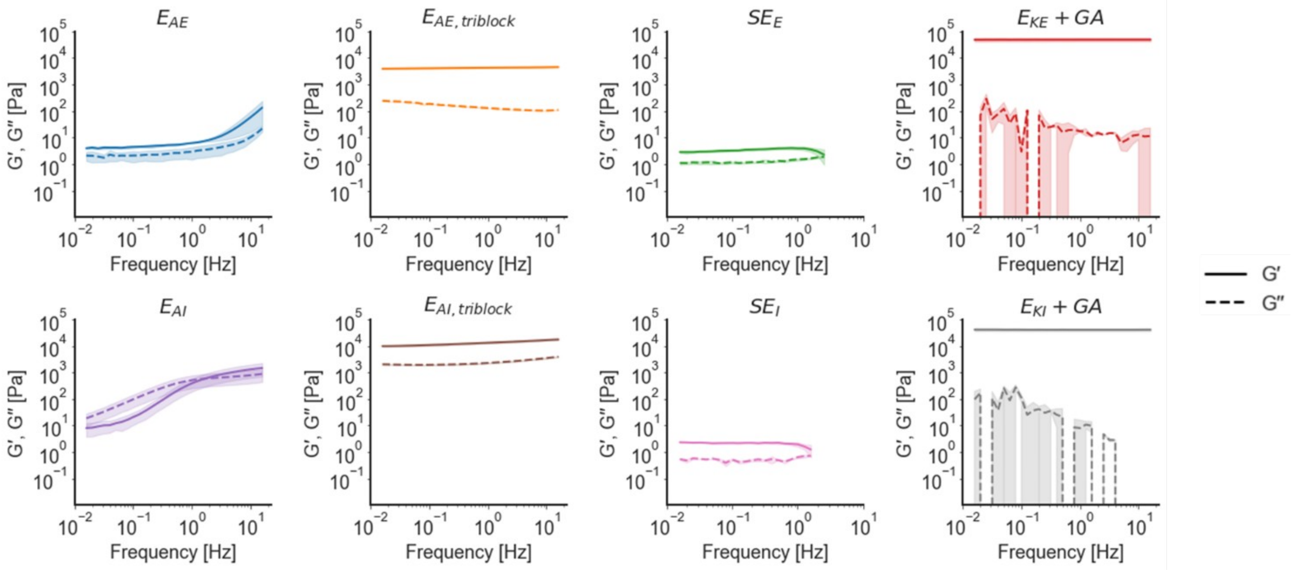

***Figure S15** – Frequency sweeps (f=0.01-15 Hz, γ=0.3%.) of ELP solutions (15 wt % in milliQ water, with a 40:1 ELP:glutaraldehyde ratio for E_{KE} and E_{KI}) (n=2). The high-frequency data for SE_E and SE_I was dominated by inertia and was left out of the plot.*

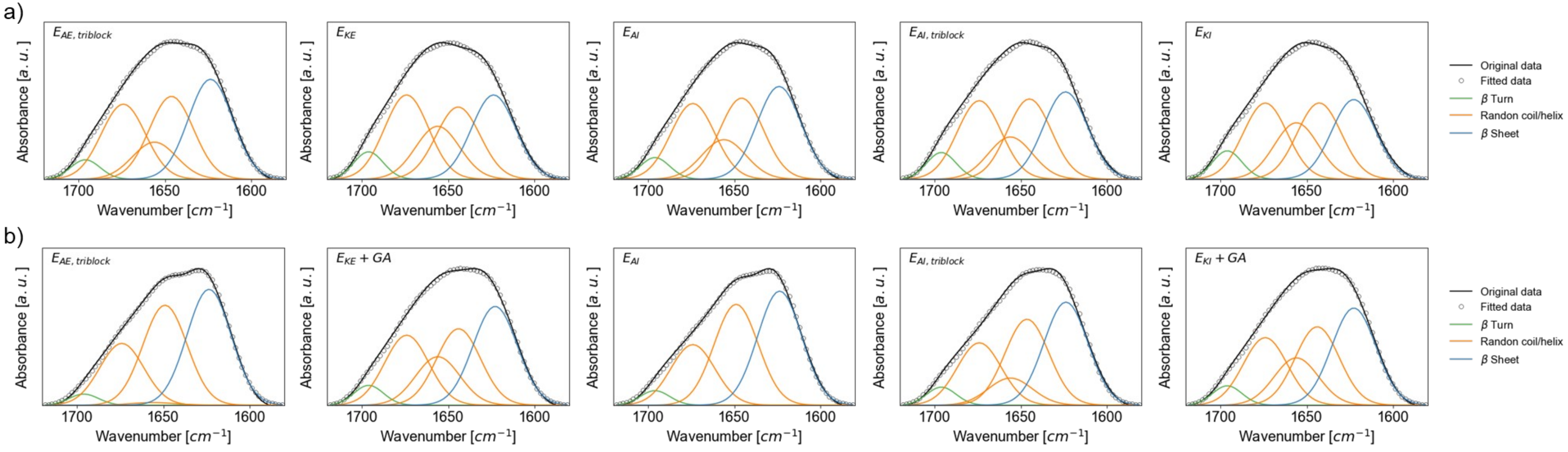

***Figure S16** – Deconvolution of the amide I spectra for freeze-dried and cryo-fractured ELP solutions (15 wt % in milliQ water, with a 40:1 ELP:glutaraldehyde ratio for E_{KE} and E_{KI}) (top row) and ELP chemical (+GA) or physical hydrogels (bottom row) to quantify their secondary structure (n=2).*

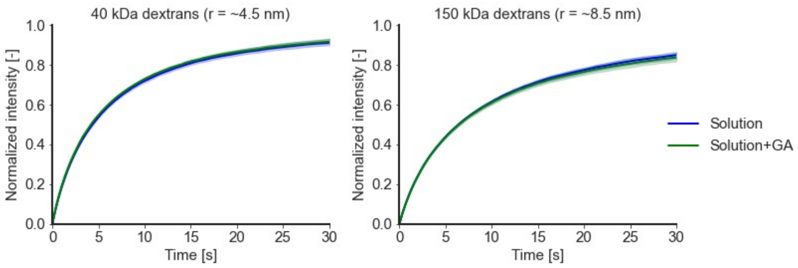

Figure S17 *– Normalized FRAP recovery curves for control samples consisting of 1 mg/mL of FITC-dextrans (40 and 150 kDa) in milliQ water ("Solution") and in milliQ water and glutaraldehyde ("Solution+GA") used to form chemical hydrogels (n=5).*

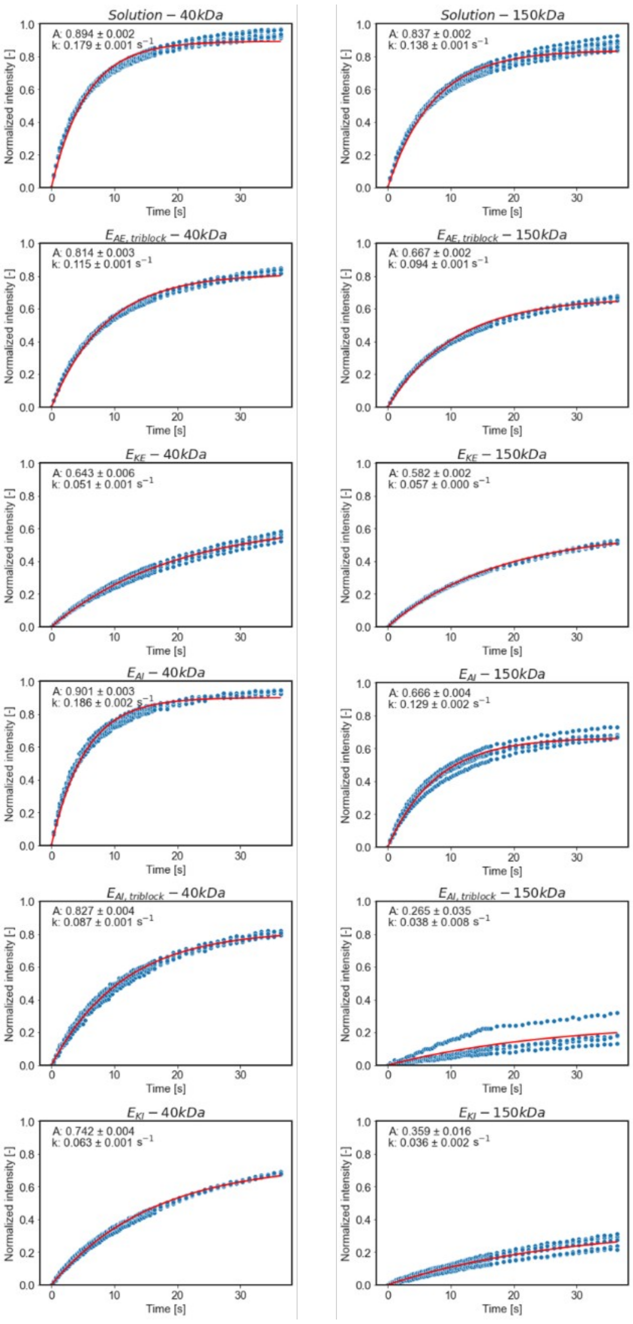

Figure S18 *– Fitting of the normalized FRAP recovery curves (blue symbols) using a single exponential curve (red lines) (n=5). The fitting parameters for each case and their error as obtained from the fit are shown in each subplot. The parameter A [unitless] corresponds to the plateau intensity after fluorescence recovery, whereas k_{FRAP} is the fluorescence recovery rate [s^{-1}].*

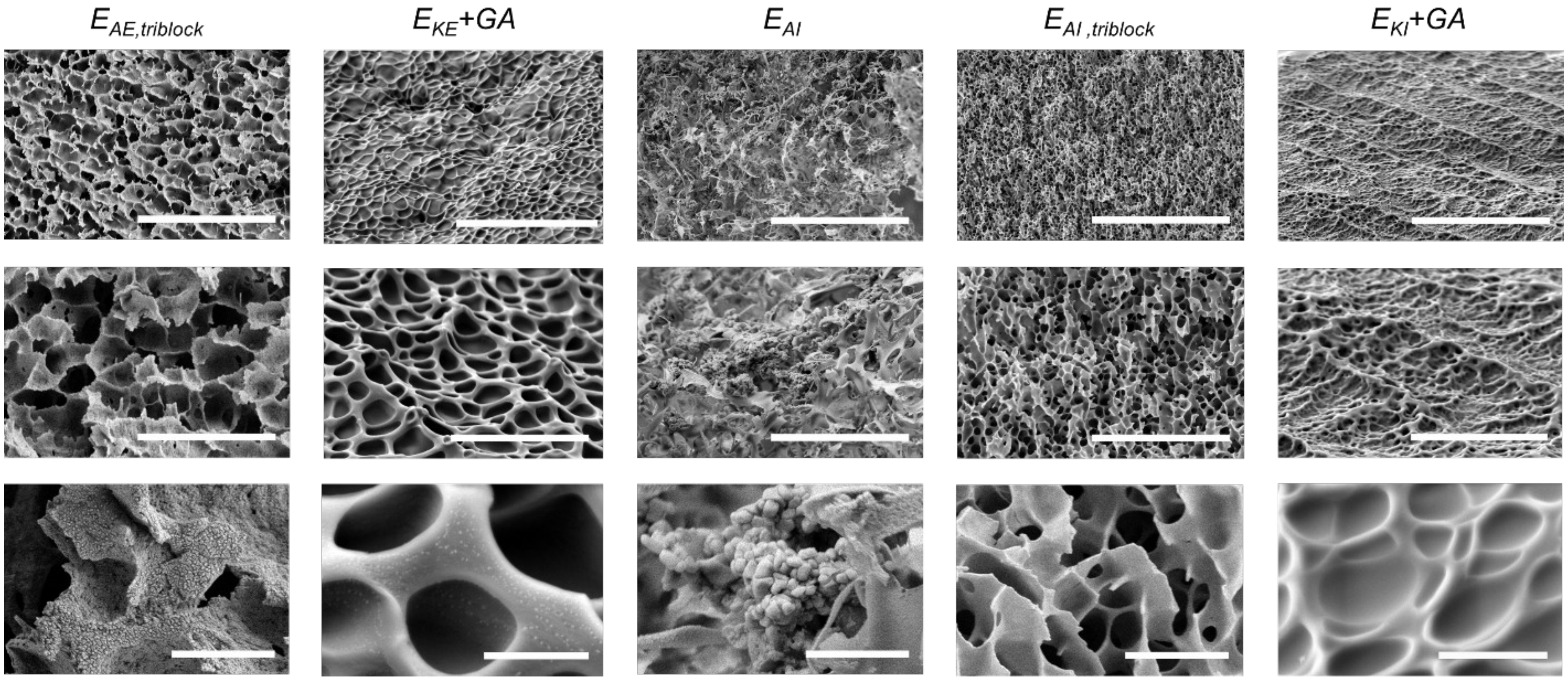

Figure S19 *– SEM micrographs of cryo-fractured samples from physical ($E_{AE,triblock}$, E_{AI}, and $E_{AI,triblock}$) and chemical hydrogels (E_{KE}+GA and E_{KI}+GA). Scale bar is 50 µm (top row), 20 µm (central row), and 3 µm (bottom row).*

REFERENCES

1. *Rodríguez-Cabello, J. C., Girotti, A., Ribeiro, A. & Arias, F. J. Synthesis of genetically engineered protein polymers (recombinamers) as an example of advanced self-assembled smart materials. Methods Mol. Biol. 811, 17–38 (2012).*
